# Transcallosal white matter and cortical gray matter variations in autistic adults aged 30–73 years

**DOI:** 10.1186/s13229-025-00652-6

**Published:** 2025-03-06

**Authors:** Young Seon Shin, Danielle Christensen, Jingying Wang, Desirae J. Shirley, Ann-Marie Orlando, Regilda A. Romero, David E. Vaillancourt, Bradley J. Wilkes, Stephen A. Coombes, Zheng Wang

**Affiliations:** 1 Laboratory for Rehabilitation Neuroscience, Department of Applied Physiology and Kinesiology, University of Florida, PO Box 118206, Gainesville, FL 32611-8205 USA; 2 Neurocognitive and Behavioral Development Laboratory, Department of Applied Physiology and Kinesiology, University of Florida, PO Box 118206, Gainesville, FL 32611-8205 USA; 3 Center for Autism and Related Disabilities (CARD), University of Florida, Gainesville, FL 32606 USA; 4 UF Health Center for Autism and Neurodevelopment (UF Health CAN), University of Florida, Gainesville, FL 32606 USA; 5 Department of Psychiatry, University of Florida, Gainesville, FL 32606 USA; 6 Department of Neurology and McKnight Brain Institute, University of Florida, Gainesville, FL 32610 USA; 7 Department of Biomedical Engineering, University of Florida, Gainesville, FL 32611 USA; 8 University of Florida, PO Box 118205, Gainesville, FL 32611-8205 USA

**Keywords:** Autism spectrum disorder, Transcallosal tracts, White matter, Gray matter, Free water, Free water corrected fractional anisotropy, Free water corrected mean diffusivity, Diffusion MRI, Autistic adults, Aging

## Abstract

**Background:**

Autism spectrum disorder (ASD) is a lifelong condition that profoundly impacts health, independence, and quality of life. However, research on brain aging in autistic adults is limited, and microstructural variations in white and gray matter remain poorly understood. To address this critical gap, we assessed novel diffusion MRI (dMRI) biomarkers, free water, and free water corrected fractional anisotropy (fwcFA), and mean diffusivity (fwcMD) across 32 transcallosal tracts and their corresponding homotopic grey matter origin/endpoint regions of interest (ROIs) in middle and old aged autistic adults.

**Methods:**

Forty-three autistic adults aged 30–73 and 43 age-, sex-, and IQ-matched neurotypical controls underwent dMRI scans. We examined free water, fwcFA, fwcMD differences between the two groups and age-related pattern of each dMRI metric across the whole brain for each group. The relationships between clinical measures of ASD and free water in regions that significantly differentiated autistic adults from neurotypical controls were also explored. In supplementary analyses, we also assessed free water uncorrected FA and MD using conventional single tensor modeling.

**Results:**

Autistic adults exhibited significantly elevated free water in seven frontal transcallosal tracts compared to controls. In controls, age-related increases in free water and decreases in fwcFA were observed across most transcallosal tracts. However, these age-associated patterns were entirely absent in autistic adults. In gray matter, autistic adults showed elevated free water in the calcarine cortices and lower fwcMD in the dorsal premotor cortices compared to controls. Lastly, age-related increases in free water were found across all white matter and gray matter ROIs in neurotypical controls, whereas no age-related associations were detected in any dMRI metrics for autistic adults.

**Limitations:**

We only recruited cognitively capable autistic adults, which limits the generalizability of our findings across the full autism spectrum. The cross-sectional design precludes inferences about microstructural changes over time in middle and old aged autistic adults.

**Conclusions:**

Our findings revealed increased free water load in frontal white matter in autistic adults and identified distinct age-associated microstructural variations between the two groups. These findings highlight more heterogeneous brain aging profiles in autistic adults. Our study also demonstrated the importance of quantifying free water in dMRI studies of ASD.

**Supplementary Information:**

The online version contains supplementary material available at 10.1186/s13229-025-00652-6.

## Introduction

Autism spectrum disorder (ASD) is an increasingly prevalent neurodevelopmental disorder characterized by atypical brain morphological variations across the lifespan [1–4]. Studies in autistic children have consistently identified the corpus callosum as a critical white matter structure that underpins many key clinical features of ASD (for a review, see [5]). Meta-analyses of diffusion MRI (dMRI) studies have reported reduced fractional anisotropy (FA) and increased mean diffusivity (MD) and radial diffusivity (RD) in the corpus callosum of autistic children, indicating underdeveloped inter-hemispheric integrity and delayed myelination during early development [6–8]. Such alterations, along with evidence of reduced structural connectivity in long range intra-hemispheric tracts, support the *underconnectivity hypothesis* in ASD, which posits a reduced capacity for information integration within large-scale cortical networks [9, 10] (although increased connectivity has been observed in some local and short-range cortico-subcortical regions, see [11]). For gray matter, studies of young autistic individuals predominantly reported volumetric deviations in the frontal and parietal lobes, the limbic system, and periventricular regions [12–16]. However, the direction and extent of these volumetric changes vary across studies, highlighting the heterogeneous nature of ASD and its complex implications for brain morphology during development. Despite considerable research in early development, remarkably little is known about microstructural variations in the white and gray matter of autistic adults as they age. The considerable lack of structural imaging studies in mid-life and older autistic adults hinders an understanding of brain aging across the adult lifespan in ASD.

Advancements in diffusion MRI (dMRI) have greatly enhanced our knowledge of axonal architecture and neuronal integrity in vivo [17–21]. Among these advancements, a novel dMRI metric known as free water has garnered significant attention in studies of neurodegenerative conditions and demonstrates promise in distinguishing pathological aging and monitoring disease progression [22, 23]. *Free water* refers to unconfined water molecules that move without directional constraints of surrounding cellular structures (i.e., neurons and axons) [24–26]. With age, free water tends to accumulate in the extracellular space of the ventricles and brain parenchyma due to various structurally degenerative processes, including neuroinflammation, cytotoxicity, neuronal damage, and axonal shrinkage [17–19, 27, 28]. Given that epidemiological studies have identified high rates of comorbid neurodegenerative diseases in ASD [29–33] and increased free water demonstrates itself to be a hallmark of brain aging [17–19], middle and old aged autistic adults may show a greater propensity for elevated free water compared to neurotypical controls.

Localized increases in free water have been documented in ASD. For example, a large cohort study of autistic children aged 9–16 years identified elevated free water in multiple cortical-basal ganglia tracts, with free water in the bilateral dorsolateral prefrontal cortex-to-caudate tracts associated with more pronounced restricted and repetitive behaviors [34]. Another study demonstrated that higher free water in the hippocampal formation at baseline predicted accelerated declines in long-term visual memory over a 2–4 year period in middle aged autistic adults [35]. Furthermore, studies using free water corrected dMRI metrics revealed significantly lower free water corrected MD (fwcMD) in nearly all brainstem tracts of autistic children aged 6–11 years, and was strongly linked to sensory hypo-responsiveness and tactile responsivity [36]. These seminal findings highlight free water accumulation in specific brain regions throughout the lifespan of ASD. Together, free water and free water-corrected scalar metrics offer critically nuanced microstructural quantifications of white and gray matter alterations in autistic individuals.

Our study expands upon existing literature by examining free water, free water-corrected FA (fwcFA), and fwcMD in 32 transcallosal white matter tracts and their corresponding homotopic gray matter origins/endpoints in autistic adults and neurotypical controls. While conventional dMRI metrics have been commonly reported in prior studies of ASD, we chose to examine free water due to its utility in characterizing brain aging and neurodegeneration. Moreover, free water-corrected dMRI metrics offer more accurate microstructural quantifications in brain regions adjacent to cerebrospinal fluid [27, 37], many of which are implicated in ASD. By concurrently examining homologous white and gray matter regions of interest (ROIs), we were able to assess distinct age-related variations in areas presumed to share similar microstructural profiles. This approach also allowed us to address whether age-associated increases in free water would simultaneously present in these white and gray matter regions in ASD.

Prior research in middle and old aged autistic adults has reported comparable FA, MD, and RD in the corpus callosum between autistic adults and controls [38, 39]. Given the limited evidence from free water imaging studies in ASD and lack of significant findings from conventional dMRI metrics in autistic adults, we hypothesized that autistic adults would exhibit minimal differences in fwcFA and fwcMD but show increased free water in transcallosal tracts compared to neurotypical controls. We extended this hypothesis to gray matter, despite being the first study to quantify free water, fwcFA, and fwcMD in gray matter regions in autistic adults. Additionally, we examined age-associated patterns in free water and free water-corrected dMRI metrics in both autistic adults and neurotypical controls. Building on sparse dMRI studies in this age range [39], we predicted that autistic adults would exhibit more pronounced reductions in fwcFA but increases in free water and fwcMD in old age. Lastly, we explored associations between clinical measures of ASD and free water in regions that significantly differentiated autistic adults from controls. To align with the majority of prior dMRI studies and evaluate the effect of free water correction, we also extracted uncorrected FA and MD using conventional single tensor modeling in the same white and gray matter ROIs for both groups. The findings are available in the supplementary materials section.

## Materials and methods

All study procedures were approved by the University of Florida (UF) Institutional Review Board following the Declaration of Helsinki. The IRB number is 202,100,659, with an approval date of July 26, 2022.

### Study participants

Forty-three autistic adults and 43 neurotypical controls participated in this study. Participants were between 30 and 73 years and groups were matched on age, sex, and IQ (Table 1). Autistic adults were identified and recruited from the Center for Autism and Related Disabilities (CARD) at the University of Florida in Gainesville, the University of Central Florida, the University of South Florida, as well as the SPARK Research Match program (https://www.sfari.org/resource/research-match/). Neurotypical controls were recruited primarily from communities in North-Central Florida through study flyers and word of mouth. All participants provided written informed consent after receiving a complete description of the study.

**Table 1:** Demographic and clinical characteristics and dMRI quality between autistic adults (ASD) and neurotypical controls (NT)

	ASD mean (± SD)	NT mean (± SD)	t/χ^2^	*p*
Age (years)	47.21 (± 10.86)	49.79 (± 12.01)	− 1.05	0.299
Range	30–73	30–70	–	–
Sex (M/F)^a^	25/18	23/20	0.19	0.664
Handedness (R/L/B)^a/b^	39/3/1	39/4/0	1.14	0.565
Full-scale IQ	107.44 (± 13.71)	107.87 (± 11.02)	− 0.16	0.877
Verbal IQ	107.72 (± 13.78)	106.21 (± 11.23)	0.54	0.589
Non-verbal IQ	105.33 (± 13.90)	107.90 (± 13.49)	− 0.85	0.399
ADOS-2	10.72 (± 3.31)	N/A	–	–
RBS-R	44.44 (± 26.88)	3.28 (± 3.73)	21.21	< 0.001***
Total brain volume (cm^3^)	1555.34 (± 161.11)	1515.17 (± 146.34)	1.21	0.230
Head motion (mm)	1.13 (± 1.03)	0.75 (± 0.50)	2.17	0.034*

^a^Chi-square (χ^2^) statistics

^b^Self-reported handedness: R = right-hand dominant, L = left-hand dominant, and B = ambidextrous

Total raw scores were reported for ADOS-2 and RBS-R

Statistical significance is bold-faced and labeled with *p < 0.05, ***p < 0.001

Prospective autistic adults with a clinical diagnosis of ASD were screened using the Autism Spectrum Quotient for Adults (AQ) [40] and the Social Responsiveness Scale Adult Self-Report (SRS-2) [41]. The AQ comprises five sub-scales that evaluate individuals’ social skill, attention switching, attention to detail, communication skill, and imagination. The SRS-2 includes subscale assessments on social awareness, social cognition, social communication, social motivation, restricted interest, and repetitive behavior. Individuals who scored > 32 on the AQ and ≥ 65 on the SRS-2 were invited to receive a diagnostic evaluation using the Autism Diagnostic Observation Schedule, Second Edition (ADOS-2) [42] at the UF CARD. Diagnosis for autistic adults was confirmed through a comprehensive review of AQ, SRS-2, ADOS-2, and expert clinical opinion following the DSM-5 criteria [43]. Three autistic adults did not meet the cut-off for AQ or SRS-2 but scored > 7 on ADOS-2. Their diagnosis was later confirmed by research reliable clinicians (AMO and RAR) on our team. Autistic adults were excluded if they had a known genetic or metabolic disorder associated with ASD (e.g., Fragile X syndrome, Rett syndrome, Phelan McDermid syndrome, tuberous sclerosis).

Prospective controls who scored ≤ 22 on the AQ and < 60 on the SRS-2 were recruited. Prospective controls were excluded if they reported a family history of ASD or other neurodevelopmental disorders in their first- and second-degree relatives. All prospective participants who met any of the following criteria were excluded from the study: (1) confirmed diagnosis of intellectual disability, mild cognitive impairment, or dementia; (2) confirmed diagnosis of non-specific developmental delay; (3) recent history of or current major psychiatric conditions (e.g., schizophrenia, bipolar disorder or post-traumatic stress disorder); (4) recent history of or current medical illness that significantly affects the structure and/or function of the central nervous system (e.g., brain tumor, thyroid disease, Cushing’s disease, or HIV infection); (5) confirmed diagnosis of a neurological disorder (e.g., stroke, dystonia, seizure disorders, Parkinson’s disease, or cerebellar ataxia); (6) family history of a hereditary neurological disorder (e.g., Huntington’s Chorea, Wilson’s Disease, or amyotrophic lateral sclerosis); (7) wearing implanted medical devices (e.g., pumps, cardiac pacemakers, or cochlear implants); (8) pregnant; (9) had a full-scale IQ (fs-IQ) < 75, or 10) non-English speaking.

During initial intake, one autistic adult reported birth asphyxia, while two others reported prolonged delivery at birth. Additionally, nine autistic adults disclosed a history of concussion, either due to risky play during childhood or car collisions in adulthood. To ensure participant safety, we did not require individuals to withhold routine psychotropic medications for the MRI sessions. Psychotropic medication taken 48 h prior to testing included antipsychotics (ASD = 4), mood stabilizers (ASD = 2), stimulants (ASD = 7), antidepressants (ASD = 26, NT = 4), and sedatives (ASD = 6). As part of the study, all participants completed the Repetitive Behavior Scale-Revised (RBS-R) [44] and had their IQ assessed using the Wechsler Abbreviated Scales of Intelligence, 2nd Edition (WASI-II) [45].

### dMRI data acquisition

The MRI session was administered on a 3 T Siemens Prisma scanner with a 64-channel head coil at the UF McKnight Brain Institute. The dMRI images were acquired using an echo-planar imaging sequence with the following parameters: TR = 6400 ms, TE = 58 ms, voxel size = 2.0 mm × 2.0 mm × 2.0 mm, b-values: 5 × 0, and 64 × 1000 s/mm^2^, field of view = 256 × 256, number of continuous slices = 69, and bandwidth = 2442 Hx/pixel. Participants wore earplugs and headphones to minimize discomfort from instrumental noise. Head motion was restricted using foam paddings inserted around the head. The scan took 7 min and 41 s to complete.

### dMRI data post-processing and analysis

We performed post-processing and analysis of all dMRI data using a customized pipeline that combines tools from FMRIB Software Library 6.0 (FSL: https://fsl.fmrib.ox.ac.uk/; [46, 47]), Advance Normalization Tools (ANTs: https://stnava.github.io/ANTs/; [48]), and the MRtrix3 package (https://www.mrtrix.org/; [49]). First, we removed Gaussian noise present in the dMRI data by fitting a Marchenko–Pastor distribution to the signal matrices to generate a threshold for PCA denoising [49–52]. Next, we removed Gibbs-ringing artifacts that can occur at tissue borders such as the outer surface of the brain and near the ventricles [49, 53]. We then generated brain masks from dMRI images using the dwi2mask function from the MRtrix3 package, which uses information from both diffusion-weighted and non-diffusion weighted (b = 0) volumes [49, 54]. The Eddy current and movement related distortions were then corrected using FSL Eddy and gradient directions were adjusted accordingly [49, 55–58].

Consistent with prior work from our group and others [21, 25, 59], we reconstructed dMRI data using a bi-tensor model, where freely diffusing water is modeled by one tensor and anisotropic water diffusion is modeled with a separate diffusion tensor after removing the contribution from isotropic free water. In this model, the free water compartment of each voxel is interpreted primarily as originating from extracellular water diffusion and the second tensor represents the tissue compartment after removing the contribution from free water. We performed whole brain estimation of free water and calculated free water corrected tensor metrics using custom MATLAB scripts (R2023a, The Mathworks Inc., Natick, MA, USA). Briefly, we calculated free water from single shell diffusion data based on minimization of a variational regularization framework outlined in [25]. Initialization of the free water estimate in our pipeline uses MD maps calculated from a single tensor fit (i.e. Equation 5 in [60]). Thus, prior to free water estimation we performed conventional single tensor reconstruction using FSL’s DTIFIT. Next, conventional MD maps were used for initialization of the bi-tensor reconstruction in MATLAB. Voxels with MD values greater than 0.8 × $d$ (i.e., $d$ is constant diffusivity) were assumed to be comprised of corticospinal fluid and omitted from the fitting process. Because fitting a bi-tensor model with dMRI data from single diffusion data has been described as an ill-posed problem with many possible solutions, we employed sensible biological constraints to the minimization process, as in Pasternak et al. [25]. We set the reference $MD_{t}$ at 0.6 μm^2^/ms. and diffusivities were limited to $\lambda_{max}$ = 2.5 μm^2^/ms and $\lambda_{min}$ = 0.1 μm^2^/ms. We also assumed isotropic water diffusion at 37 °C as a constant ($d$ = 3.0 × 10^−3^ μm^2^/ms). After initialization, we then performed 100 iterations to refine the estimates for the free water volume fraction and free water corrected diffusion tensor that was used calculate fwcFA and fwcMD. We used an automated quality assurance procedure and visually inspected output images (e.g., FW and fwcFA) to confirm appropriate data quality.

We used ANTs to perform nonlinear registration to MNI standard space by warping each subject’s FA image to the Human Connectome Project’s (HCP) 1065 template [61]. We chose the uncorrected FA for the registration process because the HCP 1065 template was created with FA images from single tensor reconstruction. We then applied the same transformation matrix to align all images from the same subject into MNI space, which included metrics derived from the bi-tensor model (FW, fwcFA, fwcMD) and single tensor model (FA and MD).

**Fig. 1:**
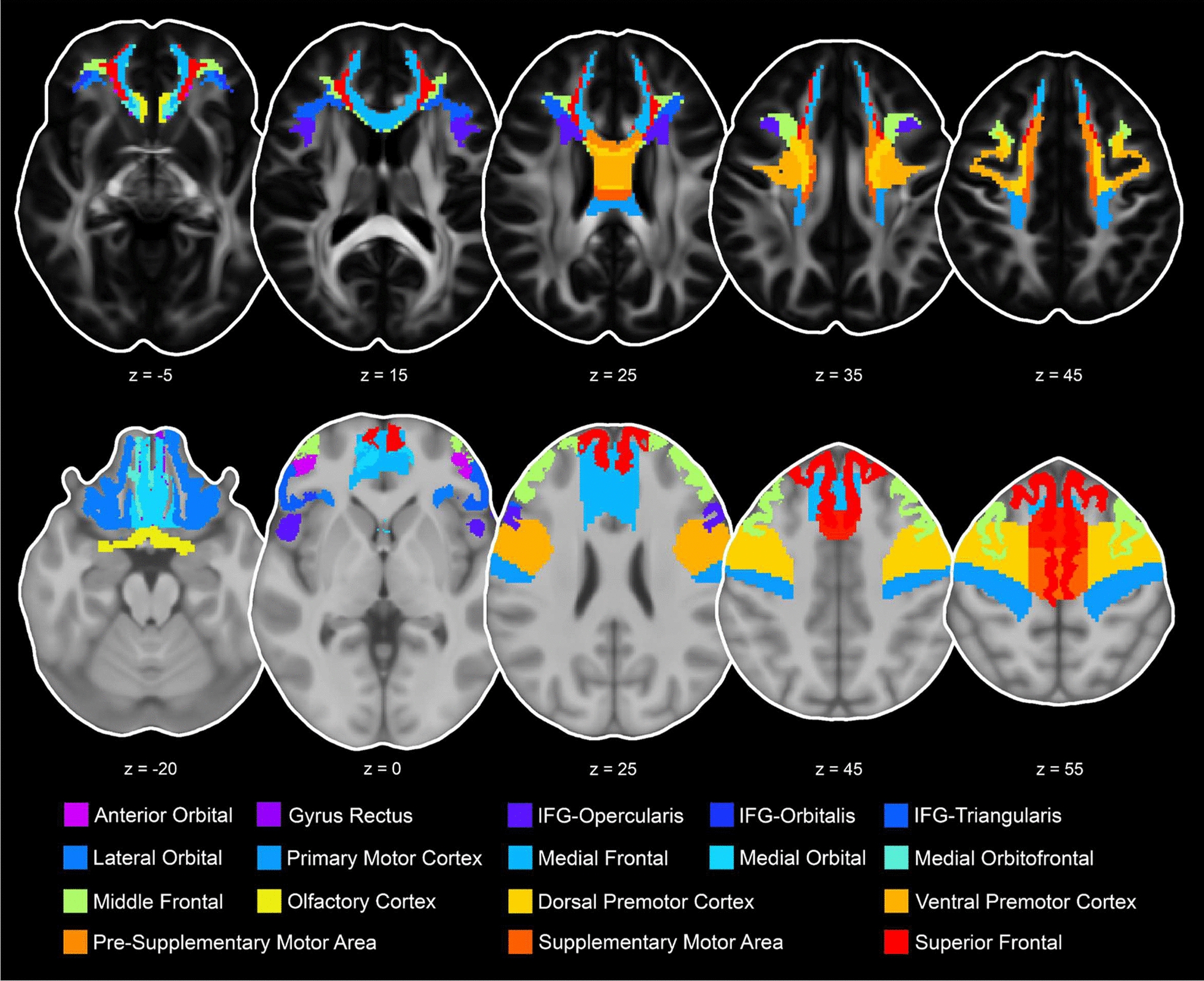
Representation of the 17 frontal transcallosal tracts overlaid on the HCP1065 FA template (top row) and gray matter origin/endpoint ROIs overlaid on the ICBM 152 T1 template (bottom row). Tracts and grey matter ROIs follow the same color code for the anterior orbital gyrus, gyrus rectus, inferior frontal gyrus-pars opercularis (IFG-Opercularis), inferior frontal gyrus-pars orbitalis (IFG-Orbitalis), inferior frontal gyrus-pars triangularis (IFG-Triangularis), lateral orbital gyrus, primary motor cortex, medial frontal gyrus, medial orbital gyrus, medial orbitofrontal gyrus, middle frontal gyrus, olfactory cortex, dorsal premotor cortex, ventral premotor cortex, pre-supplementary motor area, supplementary motor area, and the superior frontal gyrus presented in the axial view (transcallosal tract z = − 5, 15, 25, 35, 45 and gray matter z = − 20, 0, 25, 45, 55)

**Fig. 2:**
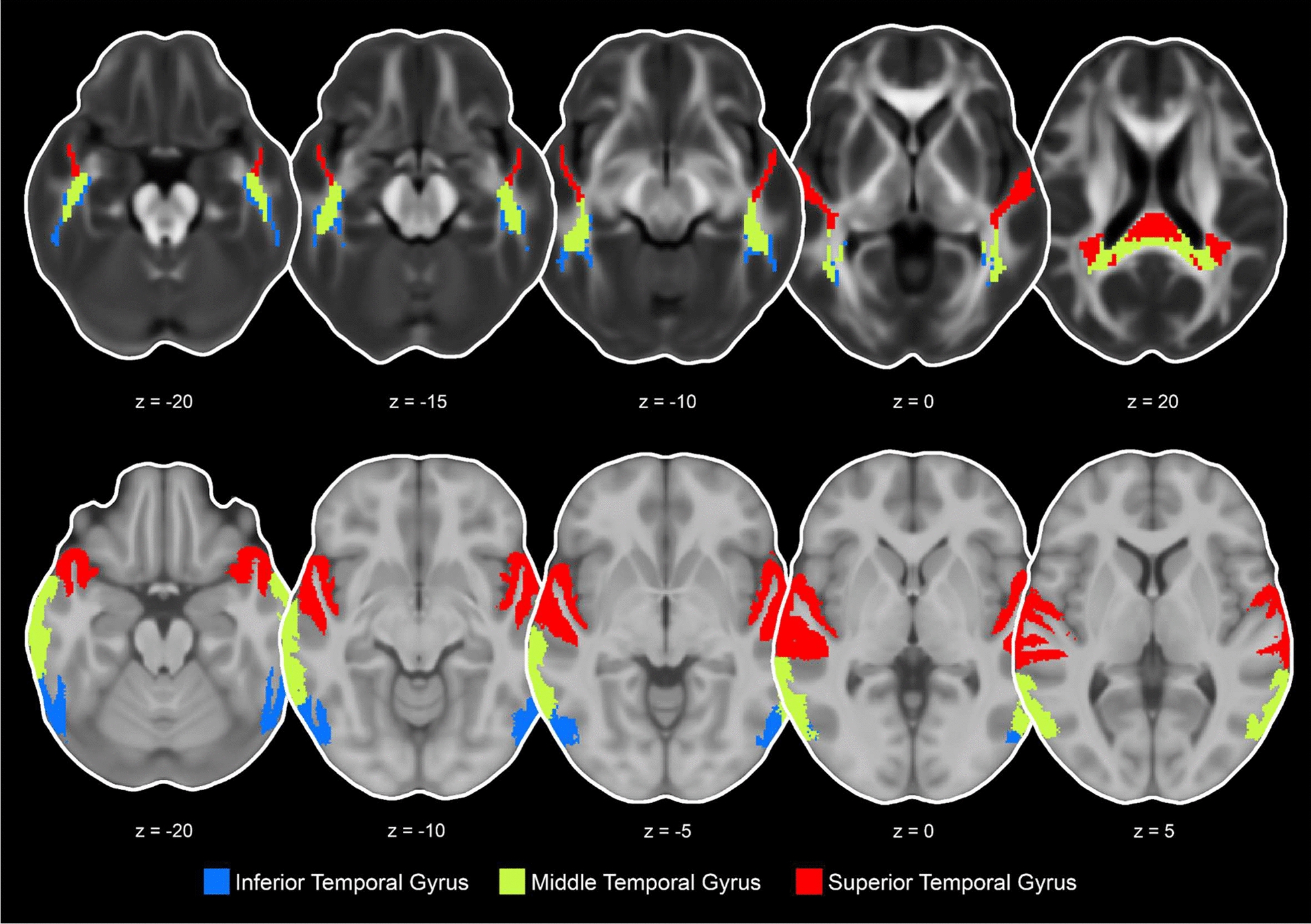
Representation of the 3 transcallosal tracts and gray matter ROIs in the temporal cortex including the inferior temporal gyrus, middle temporal gyrus, and superior temporal gyrus presented in the axial view (transcallosal tract z = − 20, − 15, − 10, 0, 20 and gray matter z = − 20, − 10, − 5, 0, 5)

**Fig. 3:**
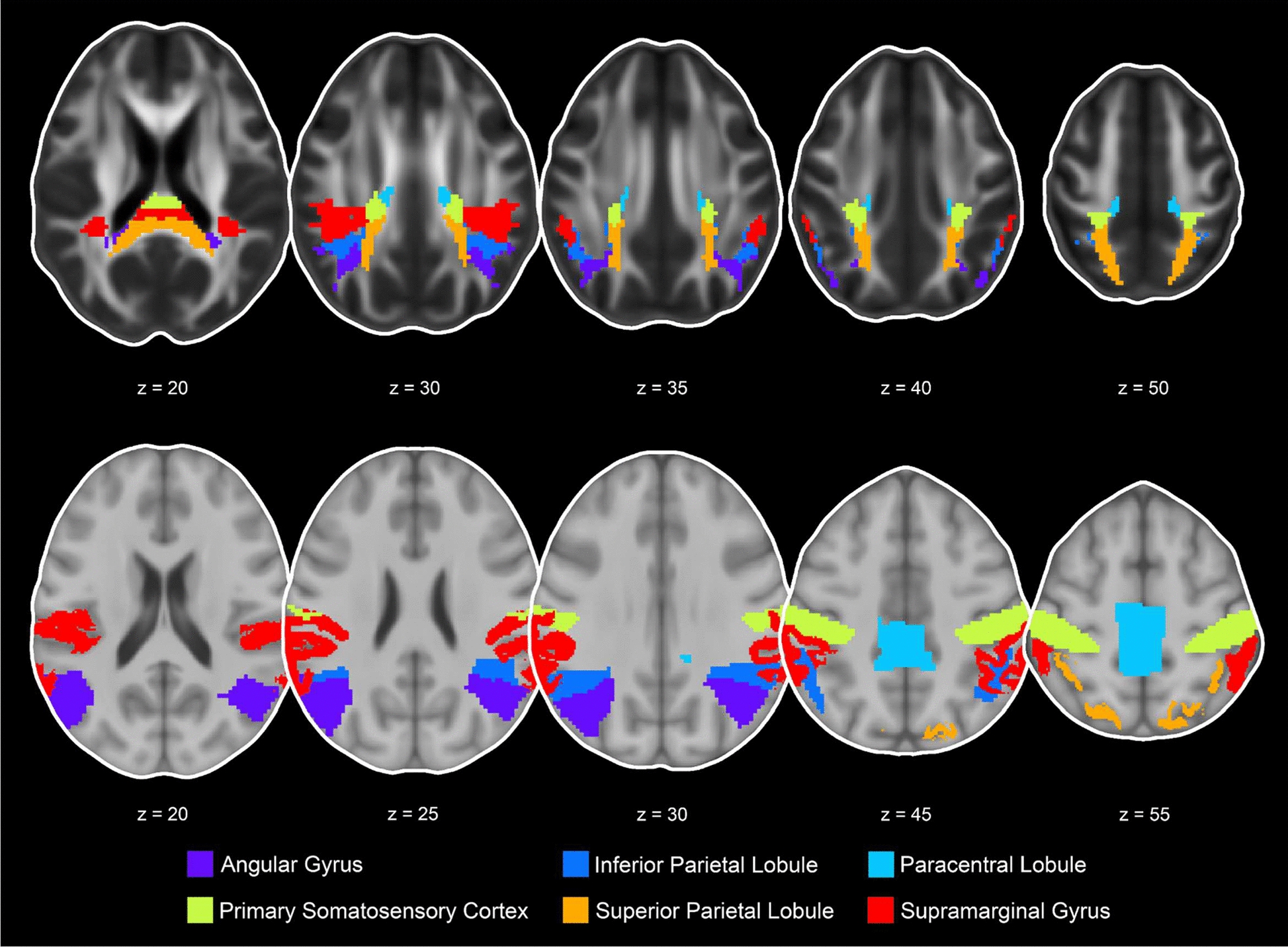
Representation of the 6 transcallosal tracts and gray matter ROIs in the parietal cortex including the angular gyrus, inferior parietal lobule, paracentral lobule, primary somatosensory cortex, superior parietal lobule, supramarginal gyrus presented in the axial view (transcallosal tract z = 20, 30, 35, 40, 50 and gray matter z = 20, 25, 30, 45, 55)

**Fig. 4:**
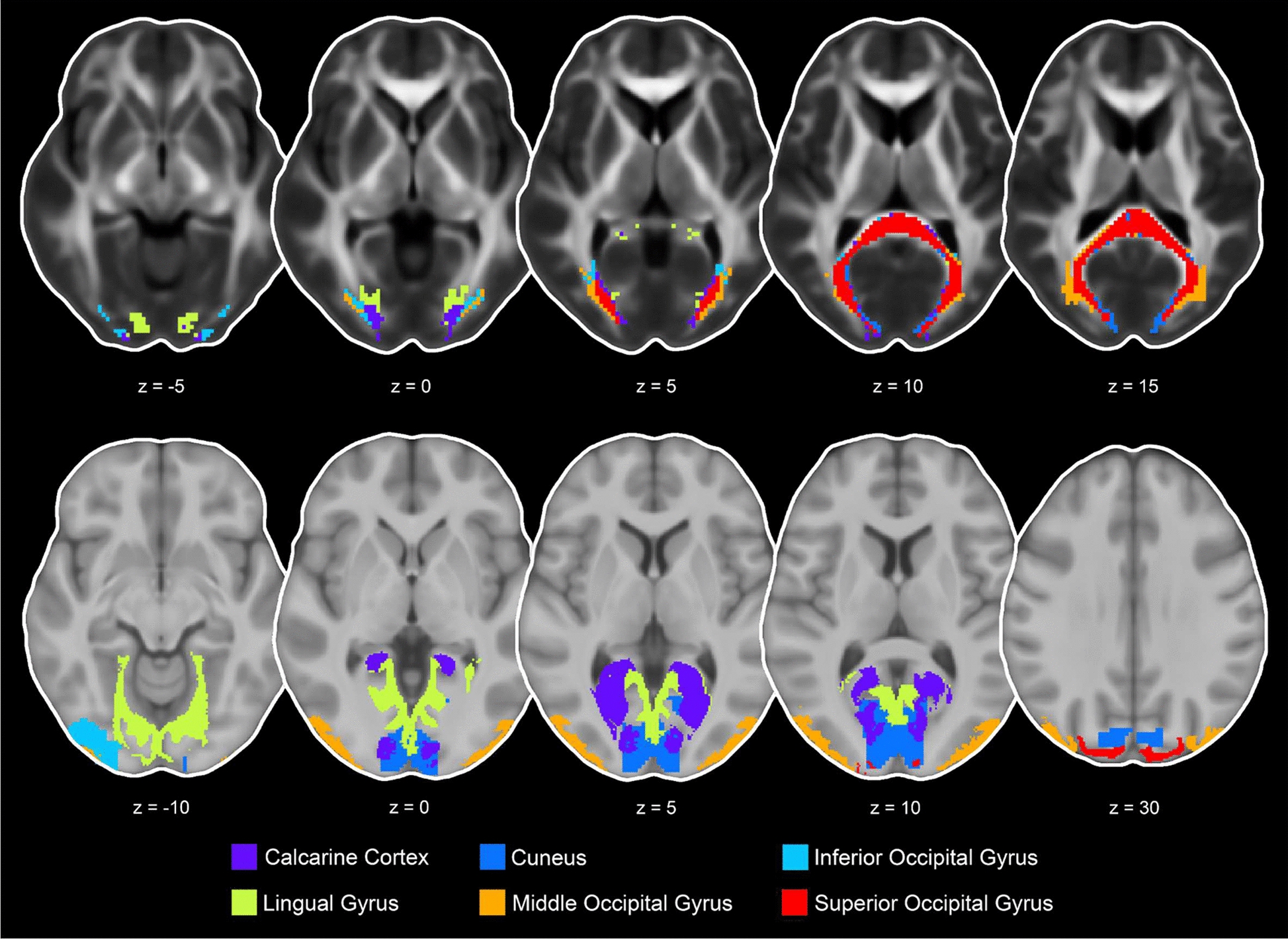
Representation of the 6 transcallosal tracts and gray matter ROIs in the occipital cortex including the calcarine cortex, cuneus, inferior occipital gyrus, lingual gyrus, middle occipital gyrus, and superior occipital gyrus presented in the axial view (transcallosal tract z = − 5, 0, 5, 10, 15 and gray matter z = − 10, 0, 5, 10, 30)

After registration to MNI space, we used regions of interest (ROIs) in MNI standard space to extract dMRI metrics of interest from the bi-tensor model (FW, fwcFA, and fwcMD). For comparison and to determine the impact of free water correction, we also extracted ROI values from dMRI metrics calculated using the conventional single tensor model (FA and MD). We utilized ROIs in white matter from the transcallosal tractography template (TCATT; [59]) and gray matter using the Mayo Clinic Adult Lifespan Template (MCALT; [62]), human motor area map (HMAP, [63]), the Desikan-Killiany atlas [64], and the Destrieux atlas [65]. The TCATT is an ROI-based template that consists of 32 commissural tracts between homotopic regions of both hemispheres in 3D. This template includes transcallosal tracts from the frontal (17), temporal (3), parietal (6), and occipital (6) cortices [59]. The MCALT was constructed from T1-weighted scans of 202 healthy controls aged > 30 years [62] making it suitable for our study given the wide age range. The Desikan-Killiany atlas and the Destrieux atlas are landmark-based templates that consist of gyral and sulcal based regions. For gray matter template, we selected 32 ROIs that correspond to the white matter TCATT template. Figures 1, 2, 3 and 4 show transcallosal white matter tracts (top row) and gray matter ROIs (bottom row) in frontal, temporal, parietal, and occipital cortices. The mean values for each dMRI metric (i.e., free water, fwcFA, fwcMD, FA, and MD) were extracted for each transcallosal tract and gray matter ROI, totaling 320 dependent variables [5 dMRI metrics × (32 transcallosal tracts + 32 Gy matter ROIs)].

### Statistical analyses

Statistical analyses were conducted using SPSS version 29 (IBM SPSS Statistics, Armonk, NY, USA) and R version 4.2.2 (https://www.R-project.org). Demographic characteristics, ASD traits, and head motion were compared between autistic adults and neurotypical controls using independent t-tests for continuous variables and Chi-square tests for categorical variables. Statistical significance was set at *p* < 0.05.

Prior to inferential statistical analysis, we applied Shapiro–Wilk test to assess the normality of dMRI variables and found that 62.5% of these measurements did not meet the assumption of normality. As a result, we implemented a one-way analysis of covariance (ANCOVA) with 5000 permutations to examine between-group differences for dMRI metrics of interest from the bi-tensor (FW, fwcFA, and fwcMD) and single tensor (FA and MD) model [66]. Each ANCOVA model included group (ASD vs. NT) as the predictor, the diffusion measure as the response variable, and age and sex as covariates. We included age and sex in the ANCOVA models due to the wide age range of our samples, and both factors have been shown to significantly affect imaging measures in autistic adults and neurotypical controls [67–69].

We conducted nonparametric partial correlation analyses with 5000 permutations to examine the effect of age on each dMRI metric separately for autistic adults and neurotypical controls [70]. Each correlation model consisted of age as the predictor, a dMRI metric as the response, and sex as the covariate [71–73]. Additionally, we performed nonparametric partial correlation analyses with 5000 permutations to examine the relationship between autistic traits and free water measures that significantly differentiated autistic adults and neurotypical controls [70]. We applied false discovery rate (FDR) corrections to control for multiple comparisons in both ANCOVA and nonparametric partial correlation analyses [74]. FDR corrections were implemented separately to each combination of dMRI metric and tissue category (e.g., fwcFA in white matter, FW in gray matter), with the q threshold set at < 0.05 [75].

## Results

Table 1 shows the demographic, clinical, and head motion comparisons between autistic adults and neurotypical controls. Both groups were matched for age, sex, self-reported handedness, IQ scores, and total brain volume. Autistic adults had significantly higher RBS-R total raw scores compared to neurotypical controls. Autistic adults also exhibited greater head motion than controls.

### Between-group comparisons across transcallosal tracts

**Fig. 5:**
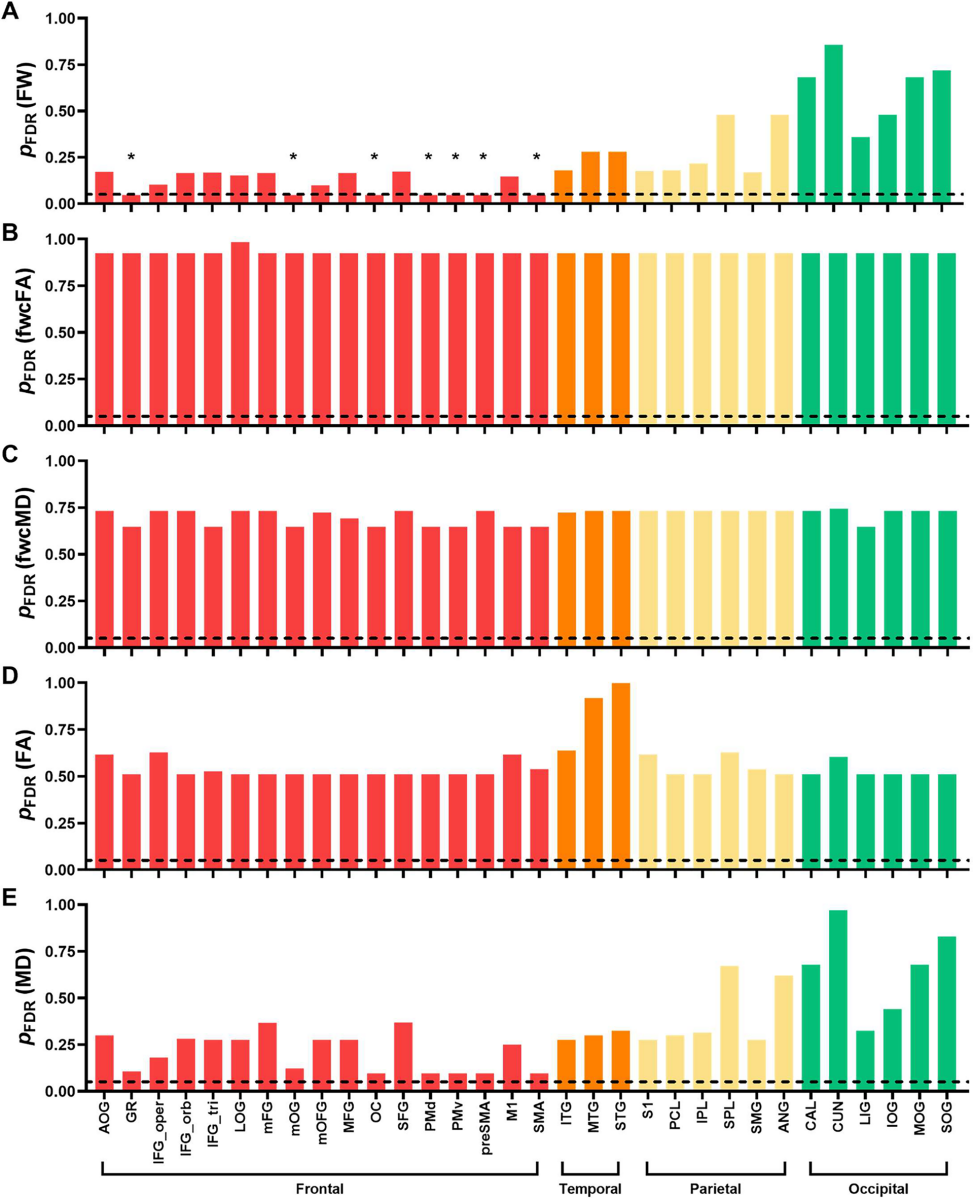
ANCOVA results for the free water (**A**), fwcFA (**B**), fwcMD (**C**), FA (**D**), and MD (**E**) across 32 transcallosal tracts in the frontal (red), temporal (orange), parietal (yellow), and occipital (green) regions. The dashed horizontal lines represent *q-*threshold of 0.05 after FDR correction. Bars below the dashed line (marked with asterisks) denote significant between-group differences, with *p*_FDR_ < 0.05

Autistic adults exhibited elevated free water in seven frontal transcallosal tracts compared to controls (Fig. 5A; Supplementary Table 1), including those connecting the gyrus rectus (GR), medial orbital gyrus (mOG), olfactory cortex (OC), dorsal premotor cortex (PMd), ventral premotor cortex (PMv), pre-supplementary motor area (preSMA), and supplementary motor area (SMA). These significant tracts are also shown together in Supplementary Fig. 1. No significant differences were found between autistic adults and controls for fwcFA or fwcMD in any transcallosal tracts after FDR correction (Fig. 5B,C; Supplementary Tables 2 and 3).

### Between-group comparisons across homotopic grey matter origin/endpoint ROIs

**Fig. 6:**
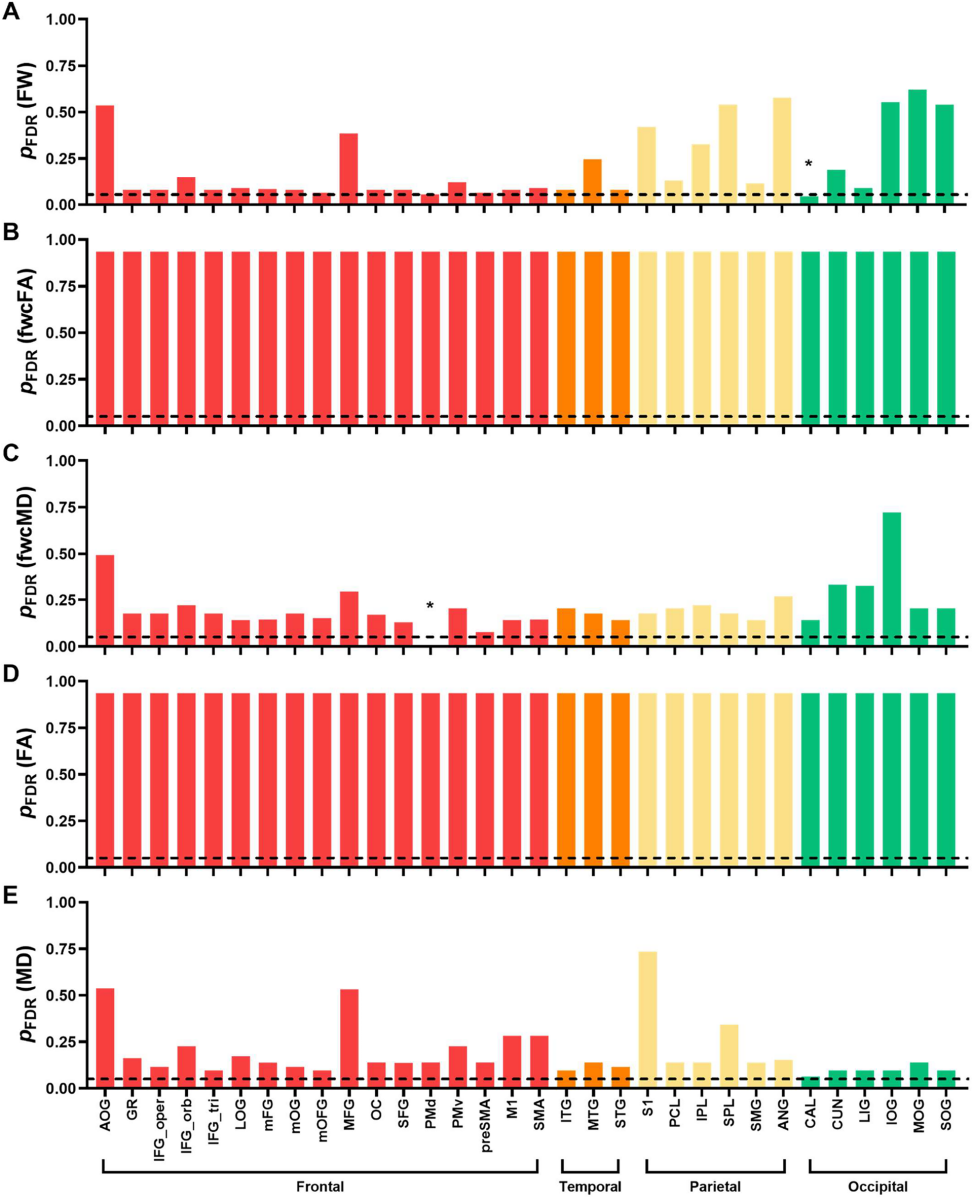
ANCOVA results for the free water (**A**), fwcFA (**B**), fwcMD (**C**), FA (**D**), and MD (**E**) across 32 homotopic grey matter origin/endpoint ROIs in the frontal (red), temporal (orange), parietal (yellow), and occipital (green) regions. The dashed horizontal lines represent *q-* threshold of 0.05 after FDR correction. Bars below the dashed line (marked with asterisks) denote significant between-group differences, with *p*_FDR_ < 0.05

Autistic adults showed elevated extracellular free water in the calcarine cortices (CAL) and lower fwcMD in PMd compared to controls (Fig. 6A,C; Supplementary Tables 4 and 6). After FDR correction, no significant differences were observed between the two groups in any gray matter ROIs for fwcFA (Fig. 6B; Supplementary Table 5).

### Age associated patterns in free water and dMRI metrics

**Fig. 7:**
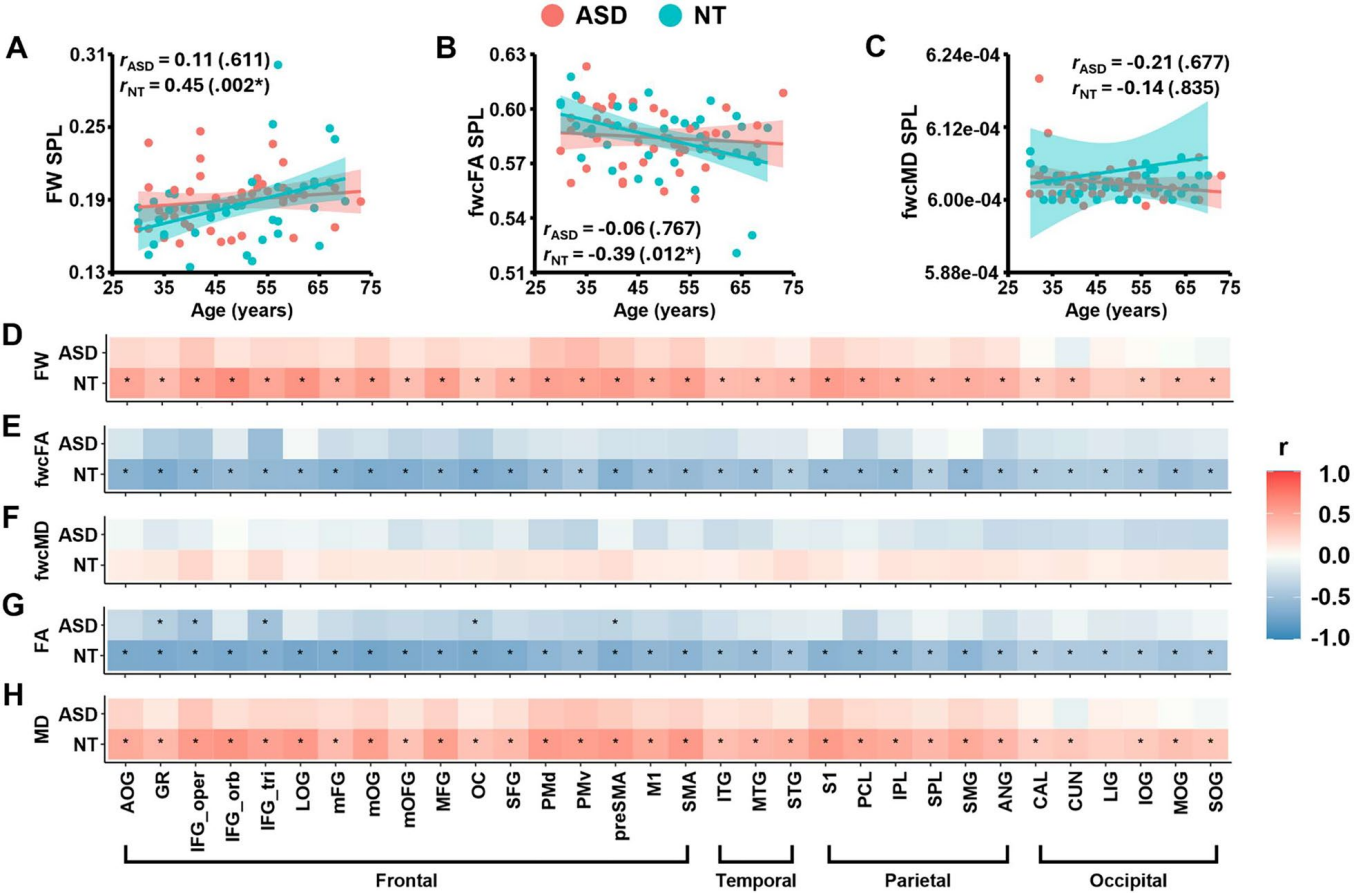
Scatterplots show free water (**A**), fwcFA (**B**) and fwcMD (**C**) of the superior parietal lobule (SPL) tract as a function of age in autistic adults (rose red) and neurotypical controls (sky blue). Panels **D**–**H** display nonparametric partial correlation results illustrating age-related effects on free water (**D**), fwcFA (**E**), fwcMD (**F**), FA (**G**), and MD (**H**) across transcallosal tracts in autistic adults and controls. The color gradient ranges from dark blue to dark red, representing r values from − 1 (strong negative correlated) to 1 (strong positive correlated). Correlations that survived FDR correction at a q- threshold of 0.05 are marked with asterisks

*Transcallosal tracts.* Fig. 7A–C show scatterplots of free water, fwcFA, and fwcMD in the superior parietal lobule (SPL) with respect to age for both groups. Across all transcallosal tracts, autistic adults exhibited distinct age-related patterns in free water and dMRI metrics compared to neurotypical controls. Specifically, control individuals showed age-related increases in free water and decreases in fwcFA across most transcallosal tracts, except for the lingual gyrus (LIG) tract, where age-related pattern for free water was not significant. In contrast, autistic adults did not exhibit any age-related patterns in free water and fwcFA (Fig. 7D,E; Supplementary Table 7). Additionally, no significant age-associated patterns were found for fwcMD in either group (Fig. 7F; Supplementary Table 7).

**Fig. 8:**
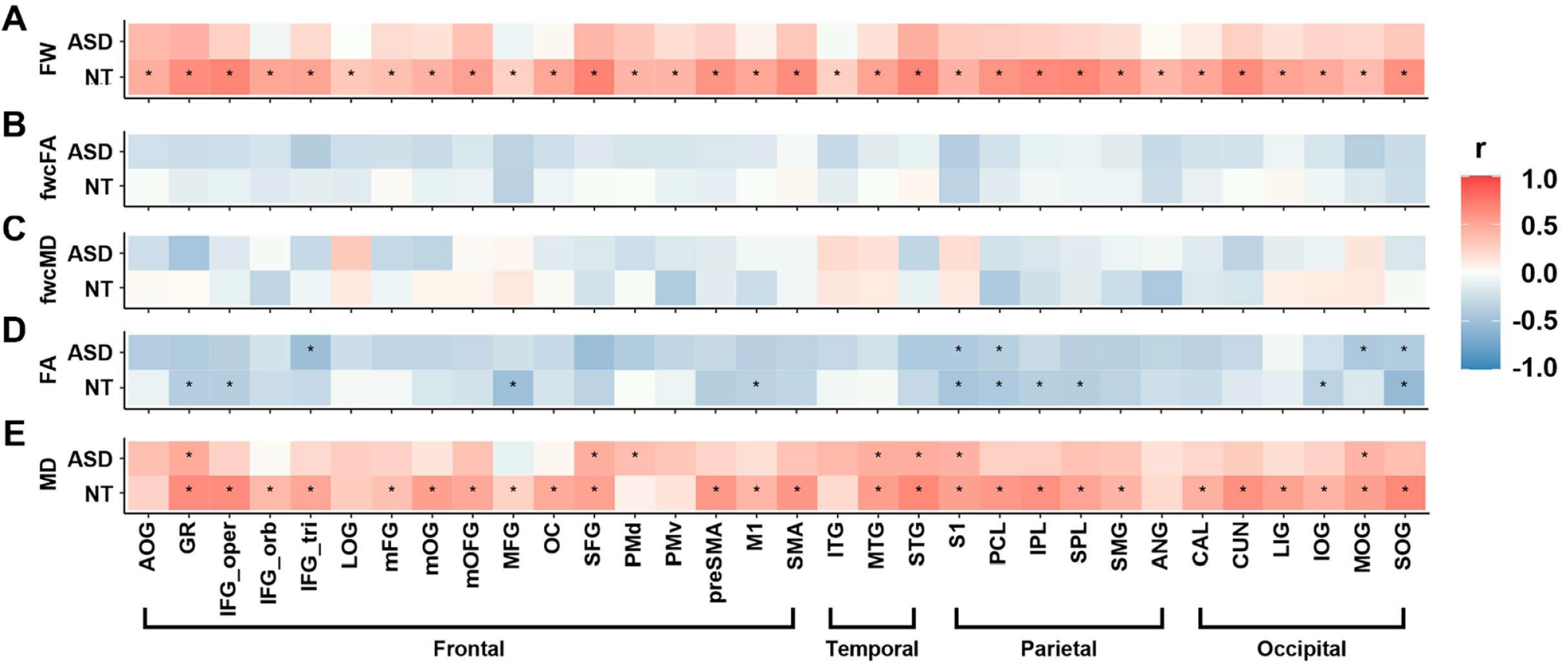
Nonparametric partial correlation results illustrate age effects on free water (**A**), fwcFA (**B**), fwcMD (**C**), FA (**D**), and MD (**E**) across homotopic grey matter origin/endpoint ROIs in autistic adults (rose red) and neurotypical controls (sky blue). The color gradient ranges from dark blue to dark red, representing r values from −1 (strong negative correlation) to 1 (strong positive correlation). Correlations that survived FDR correction at a q- threshold of 0.05 are marked with asterisks

*Homotopic gray matter regions.* Similar to observations in white matter, autistic adults exhibited strikingly different age-related patterns in free water compared to controls for gray matter (Fig. 8A; Supplementary Table 8). While neurotypical controls demonstrated age-associated increase in free water across all ROIs, autistic adults exhibited no significant relationship between free water and age. Additionally, no age-related patterns were observed for fwcFA or fwcMD in either group (Fig. 8B,C; Supplementary Table 8).

### Further inspection of age associated white and gray matter variations

**Fig. 9:**
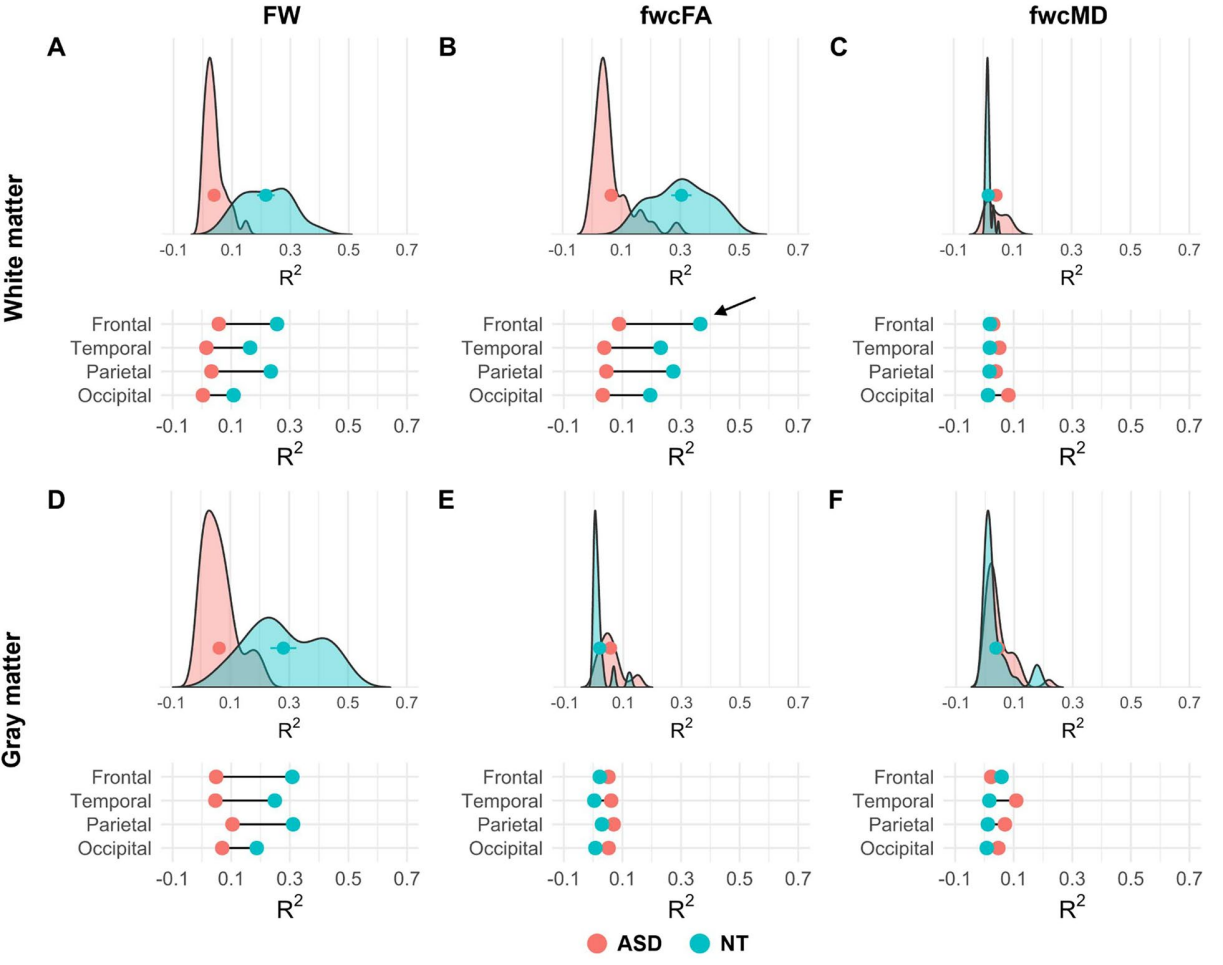
Dispersion plots of R^2^ values across 32 transcallosal tracts (top panel; **A**–**C**) and gray matter ROIs (bottom panel; **D**–**F**) of autistic adults (rose red) and neurotypical controls (sky blue). Dispersion plots for free water (**A** and **D**), fwcFA (**B** and **E**), and fwcMD (**C** and **F**) are shown from left to right. The  that sits in the center of each dispersion plot represents the [Mean + SE] of R^2^ values for each group. The [Mean] of R^2^ values derived from each of the frontal, temporal, parietal, and occipital regions are displayed at the bottom of each dispersion plot cluster and labeled by . The lines connecting these red and blue dots represent the R^2^ mean value difference between the autism and control groups

*Nonparametric partial correlation model.* We further inspected R^2^ and β coefficient values derived from nonparametric partial correlation models to identify discrete age-associated patterns in dMRI metrics for white and gray matter in autistic adults and neurotypical controls (Fig. 9; Supplementary Fig. 2). The R^2^ represents the goodness of fit, indicating how well a diffusion measure fits the model in relation to age. The β coefficient reflects the slope of the regression model, emphasizing the direction of change. A positive β coefficient suggests an age-associated increase in a diffusion measure, while a negative β coefficient indicates a decrease [76].

For transcallosal tracts, R^2^ values of free water (Fig. 9A) and fwcFA (Fig. 9B) were lower and tightly clustered around zero on the x-axis for the autistic group, indicating a poor fit of the linear relationship between dMRI metrics and age. In comparison, neurotypical controls showed more positive R^2^ values with a broader dispersion on the x-axis, suggesting stronger age-associated linear relationship for free water and fwcFA across transcallosal tracts. Notably, the mean R^2^ values for fwcFA in the frontal area displayed the largest between-group differences compared to those derived from the temporal, parietal, or occipital regions (black arrow in Fig. 9B). Lastly, R^2^ values and dispersions of fwcMD were largely overlapped between autistic adults and controls across all transcallosal tracts (Fig. 9C).

For gray matter, autistic adults exhibited lower R^2^ values in free water that were tightly clustered around zero on the x-axis relative to controls (Fig. 9D). Upon inspecting fwcFA and fwcMD, we observed largely overlapped R^2^ values between the two groups (Fig. 9E,F).

Supplementary Fig. 2 depicts the dispersion plots of β coefficients in autistic adults and controls. For both white and gray matter, neurotypical controls exhibited more positive β coefficients in free water compared to autistic adults. This finding indicates age-associated free water increases in controls but not in autistic individuals. Additionally, autistic adults and controls showed largely overlapped β coefficients coefficients around 0 in fwcFA and fwcMD, suggesting that there is little age-related directional pattern in both groups for these metrics.

*Nonlinear regression model.* A nonlinear regression model was employed in a follow-up analysis to further explore the age effect on free water and dMRI metrics in both groups (Supplementary Tables 9 and 10). This approach was used for two key reasons: (1) the linear regression models demonstrated a lack of fit in quantifying age-associated variations in white and gray matter for autistic adults (Supplementary Tables 9 and 10) and (2) previous studies have identified quadratic relationships in structural imaging metrics with age among younger autistic individuals [14, 77]. The nonparametric quadratic model included age and age-squared as predictors, the dMRI measure as the response variable, and sex as a covariate. Across dMRI metrics (free water, fwcFA, and fwcMD), brain tissues (white vs. gray matter), and groups, age-squared only significantly predicted fwcFA in the inferior frontal gyrus-pars triangularis (IFG_tri; *p*_FDR_ = 0.039) and paracentral lobule (PCL; *p*_FDR_ = 0.015) tracts in autistic adults (Supplementary Table 9). These results suggest that quadratic age effects were not robust in predicting free water or dMRI metrics for either group. This finding reiterates our approach of using nonparametric partial correlation model to assess age effects on dMRI metrics in autistic adults and neurotypical controls.

### Free water and clinical severity in autistic adults

Autistic adults showed increased free water in 7 frontal transcallosal tracts (Fig. 5A; Supplementary Table 1). We then performed nonparametric partial correlation analyses to examine the relationship between free water in these tracts and autistic traits (Supplementary Table 11). No significant correlations were found between free water and RBS-R total raw scores in autistic adults. Higher ADOS-2 total raw scores were associated with lower free water in the GR, OC, PMd, and PMv tracts in autistic individuals, but these relationships did not survive the FDR correction.

### Supplementary analyses using conventional single tensor fitting algorithms

We also derived conventional FA and MD metrics using single-tensor fitting algorithms to align our results with most dMRI studies in ASD. After applying FDR correction, no significant between-group differences in FA or MD were identified in any white or gray matter ROIs (Figs. 5 and 6; Supplementary Tables 12–15). In neurotypical controls, we observed age-related reductions in FA and increases in MD across most transcallosal tracts, with the exception of MD in the LIG tract. In contrast, autistic adults showed no age-related patterns in MD, although five frontal tracts retained age-related reductions in FA, including the GR, inferior frontal gyrus-pars opercularis (IFG_oper), IFG_tri, olfactory cortex (OC), and preSMA tracts (Fig. 7; Supplementary Table 16). For gray matter, age-related increases in MD were globally prominent in controls, while age-associated FA reductions appeared sporadically in both groups (Fig. 8; Supplementary Table 17). Given the absence of age-related patterns in fwcFA or fwcMD for either group, age-related patterns observed in FA and MD may likely reflect partial volume effects of free water, which are not accounted for in single-tensor fitting algorithms.

## Discussion

Autism spectrum disorder (ASD) is a lifelong condition that profoundly impacts health, independence, and quality of life [1–4]. However, research on middle life and older autistic adults remains scarce, which critically hinders our understanding of brain aging in ASD. To address this pressing research gap, we employed free water and free water corrected dMRI metrics to examine microstructural variations across 32 transcallosal tracts and their corresponding homotopic gray matter origin/endpoint ROIs. We also assessed age-associated free water, fwcFA, and fwcMD variations in both groups. We report four new observations. First, autistic adults exhibited elevated free water in seven frontal transcallosal tracts and one gray matter ROI (i.e., calcarine cortices) compared to controls. Second, both groups showed comparable fwcFA or fwcMD in white and gray matter. Third, neurotypical controls demonstrated age-related increases in free water and decreases in fwcFA across most transcallosal tracts, whereas this pattern was completely absent in autistic adults. Lastly, age-related increases in free water were also evident in gray matter for controls, but no age effects were found in any dMRI metrics in autistic adults. Collectively, our findings highlight an elevated free water load in autistic adults, particularly in frontal tracts, while revealing comparable microstructural characteristics in white and gray matter between the two groups. Our results also suggest that the well-established association between age and tissue microstructure observed in neurotypical controls is absent in autistic adults, who instead exhibited more heterogeneous microstructural deviations with age.

### Elevated free water in autistic adults

We observed increased free water in seven frontal transcallosal tracts and the calcarine cortices in middle and old aged autistic adults, suggesting that elevated free water more prominently affects white matter in ASD. Supporting this observation, a previous study using neurite orientation and dispersion imaging (NODDI) identified increased isotropic volume fraction (ISOVF) in the posterior commissural fibers in autistic adults aged 19–52 years, linking higher ISOVF values also to elevated extracellular free water in autistic individuals [78]. Although dMRI presents limitations in precisely pinpointing specific neurobiological processes or microstructural variations, our findings, combined with various lines of evidence from other studies, suggest that neuroinflammation may play a significant role in the observed free water accumulation in white and gray matter in ASD.

For instance, postmortem studies have reported elevated neuroinflammatory processes in autistic individuals, characterized by heightened activation of astroglia and microglia, as well as elevated levels of cytokines and chemokines in the brain, including IL-6, TGF-β_2, IFN-γ, and MCP-1 [22, 23, 79–81]. Preclinical studies using transgenic rat models of ASD have also demonstrated increased blood–brain barrier permeability, influencing inflammatory mediators and immune cells to translocate from the peripheral blood into the brain [82]. These mediators and cells promote inflammatory processes in the interstitial extraneuronal space [22, 23, 79–81] ultimately leading to free water accumulation [82, 83]. Furthermore, a recent preclinical study in wild-type mice found significant associations between increased free water and elevated IFN-γ levels following ventricular injection of IFN-γ at 1, 5, and 8 months [83], offering direct evidence supporting neuroinflammation in driving free water accumulation in the brain.

Increased free water has been consistently reported during prodromal or early onset stages of several neurodegenerative conditions [83–86], suggesting that neuroinflammation precedes microstructural tissue damage and emphasizes free water as a promising predictive biomarker for pathological aging [21, 87, 88]. Given that autistic adults face an elevated risk of neurodegenerative conditions, such as parkinsonism and Alzheimer’s disease [29–33], and that elevated free water has been observed in both autistic children and adults, future research should investigate the impact of aging on extracellular free water and its potential as a sensitive imaging biomarker for comorbid neurodegenerative disorders in ASD.

### Negligible differences in fwcFA and fwcMD between autistic adults and neurotypical controls

Consistent with previous dMRI studies [38, 39], we did not identify between-group differences in fwcFA or fwcMD in any transcallosal tracts. This pattern was also observed in gray matter, with one exception in the dorsal premotor cortex (PMd), where autistic adults showed lower fwcMD compared to controls. Previous studies examining gray matter in middle and old aged autistic adults have yielded mixed findings. For example, Kohli et al. [89] found significant gyrification reductions and reduced cortical folding in the frontal, temporal, and parietal cortices of autistic adults, whereas Koolschijn et al. [90] reported similar gray matter outcomes across the whole brain and individual lobes between autistic adults and controls. Our study extends the existing literature by demonstrating that tissue microstructure in transcallosal white matter tracts and gray matter is generally comparable between the two groups. Future longitudinal studies are needed to monitor changes in dMRI metrics over time to better understand aging-related alterations in white and gray matter in middle and old aged autistic adults.

### Age associated variations in dMRI metrics are different between autistic adults and neurotypical controls

Despite regional variations, typical lifespan changes in white matter are characterized by increased extracellular free water and reduced FA as individuals age [18, 19, 91]. This brain aging profile is associated with various neurobiological processes, including reduced axonal density, increased synaptic loss, myelin sheath ballooning, and reduced fiber orientation coherence [92–95]. Consistent with previous research, neurotypical controls in our study demonstrated similar age-related patterns in free water and fwcFA across 32 transcallosal tracts. However, these relationships were not identified in middle and old aged autistic adults. Upon inspecting dispersion plots for white matter, we found that neurotypical controls had more positive and widely distributed R^2^ values on the x-axis for free water and fwcFA. This suggests stronger linear relationships between free water, fwcFA and age across transcallosal tracts. In contrast, R^2^ values for free water and fwcFA in the autistic group were clustered around zero, indicating a poor fit for the linear relationship but more heterogenous distributions of these dMRI metrics with respect to age in ASD. Notably, the largest between-group differences in R^2^ mean values were observed in the frontal region for fwcFA (black arrows in Fig. 9B). This finding suggests that age-related discrepancies between autistic adults and neurotypical controls are most pronounced in the frontal regions. This observation is in concert with increased free water found in the frontal tracts in autistic adults, emphasizing the heightened vulnerability of frontal areas to brain aging in ASD.

Empirical studies exploring brain morphological deviations in middle and old aged autistic adults have proposed three distinct theoretical frameworks related to aging in ASD [96, 97]. The safeguard theory hypothesizes the presence of protective neurobiological mechanisms that mitigate brain aging and degeneration in autistic adults [90]. In contrast, the double jeopardy theory posits a cumulative effect of aging on pre-existing brain deviations in ASD, which lead to accelerated decline and greater morphological deviations in later life [35, 38, 39, 98]. Meanwhile, the parallel development theory proposes typical aging trajectories in middle and old aged autistic adults relative to neurotypical controls [96, 99, 100]. Our findings in free water and fwcFA revealed heterogeneous age-related patterns in white and gray matter in autistic adults. Future studies with large sample sizes are needed to identify subgroups of autistic individuals who exhibit slower, accelerated, or typical brain aging profiles.

### Free water as a critical measure in dMRI studies of ASD

The assessment of free water and free water corrected dMRI metrics has been limited in studies of ASD [34, 35]. Previous research in autistic children and young adults has consistently shown reduced FA and increased MD and RD in the corpus callosum cross-sectionally [101–103] and longitudinally [104], supporting the notion of reduced inter-hemispheric connectivity in ASD [10, 105]. Although contradictory findings have been reported [106–109], discrepancies were mainly attributed to differences in scanner type, scan sequence, data analytical procedures (e.g., voxel-, ROI-, or tractography-based methods), demographic characteristics, or ASD heterogeneity.

One critical yet often overlooked factor in interpreting these inconsistencies is the accumulation of extracellular free water and its impact on dMRI metrics [25–27]. This is particularly relevant for white and gray matter regions adjacent to the ventricles, which are more susceptible to cerebrospinal fluid and free water exposure. Additionally, ventricular enlargement, commonly observed in autistic individuals [16, 110–112], leads to further cerebrospinal fluid accumulation, thus increasing the likelihood of biased dMRI metric estimates when conventional single tensor algorithms are applied.

Our study highlights the importance of incorporating free water quantification in dMRI studies, as we observed increased free water in seven frontal transcallosal tracts and the calcarine cortices in autistic adults. Our study also underscored free water as a sensitive brain aging biomarker for neurotypical controls, who have shown significant free water increase with age in both white and gray matter [25, 28]. After free water correction, age-associated global increases in MD were no longer present in white and gray matter for neurotypical controls (Figs. 7 and 8; Supplementary Tables 7–8). This finding highlights the role of elevated extracellular free water in driving the age effect in conventional dMRI metrics.

## Limitations and future directions

Our findings should be considered in light of several limitations. Our study only recruited cognitively capable autistic adults. Individuals with comorbid intellectual disability would need to be included in future studies to allow a comprehensive understanding of aging-associated white and gray matter variations in ASD and identify confounding variables that may accelerate pathological aging in middle and old aged autistic adults.

In addition, our study employed a cross-sectional design, while inferences about aging-related microstructural variations can only be quantified through longitudinal studies [113]. Given the lack of age associated variations in dMRI metrics in autistic adults, future longitudinal studies with a larger cohort of autistic adults are urgently needed to delineate unique longitudinal brain aging profiles in this population. This line of research could also benefit from cluster analysis to identify subgroups of autistic adults who are more vulnerable to neurodegenerative diseases versus individuals who are more resilient to pathological aging in old age [97].

Another relevant consideration is that long-term psychotropic medication exposure is known to affect brain microstructure in individuals with psychiatric conditions (i.e., schizophrenia and bipolar disorder) [114, 115]. Specifically, antipsychotics have shown to lead to drug-induced parkinsonism in some individuals [116]. Future studies should aim to quantify the impact of long-term medication use on brain morphological deviations in autistic adults and determine whether such exposure would accelerate pathological aging processes in ASD.

Finally, dMRI metrics were reconstructed using a bi-tensor free water imaging model from single shell data. Given the model constraints and initialization method used, single shell free water estimates may contain partial effects from tissue compartment MD. As a result, free water estimates are likely more reliable in white matter tracts, where MD is inherently low, while estimates in gray matter may be more influenced by tissue compartment MD [117]. Future studies deriving free water from multi-shell dMRI data will address this limitation [118].

## Conclusion

Our study is the first to comprehensively assess free water and free water corrected dMRI metrics in white and gray matter in middle and old aged autistic adults. In doing so, we report elevated free water in seven frontal transcallosal tracts as well as one gray matter ROI in autistic adults. Age-related increases in free water and decreases in fwcFA were identified across most white matter tracts in neurotypical controls, where these patterns were absent in autistic adults. Our findings highlight heterogeneous age-associated microstructural variations in white and gray matter in middle and old aged autistic adults. Future longitudinal multi-shell dMRI studies are needed to better understand trajectories of free water and free water corrected dMRI metrics across the adult lifespan in ASD.

## Supplementary Information

Supplementary Material 1.

## Data Availability

All data are available from the corresponding author upon reasonable request.

## Abbreviations

ADOS-2: Autism Diagnostic Observation Schedule, Second Edition
ANG: Angular gyrus
ANTs: Advanced normalization tools
AOG: Anterior orbital gyrus
AQ: Autism spectrum quotient
ASD: Autism spectrum disorder
CAL: Calcarine cortex
CSF: Cerebrospinal fluid
CUN: Cuneus
FA: Fractional anisotropy
FDR: False discovery rate
FW: Free water
fwcFA: Free water corrected fractional anisotropy
fwcMD: Free water corrected mean diffusivity
GR: Gyrus rectus
HMAP: Human motor area map
IFG_oper: Inferior frontal gyrus-pars opercularis
IFG_orb: Inferior frontal gyrus-pars orbitalis
IFG_tri: Inferior frontal gyrus-pars triangularis
IOG: Inferior occipital gyrus
IPL: Inferior parietal lobule
IQ: Intelligence quotient
ISOVF: Isotropic volume fraction
ITG: Inferior temporal gyrus
LIG: Lingual gyrus
LOG: Lateral orbital gyrus
M1: Primary motor cortex
MCALT: Mayo Clinic Adult Lifespan Template
MD: Mean diffusivity
MFG: Middle frontal gyrus
MOG: Middle occipital gyrus
MTG: Middle temporal gyrus
mFG: Medial frontal gyrus
mOFG: Medial orbitofrontal gyrus
mOG: Medial orbital gyrus
NODDI: Neurite orientation and dispersion imaging
NT: Neurotypical
OC: Olfactory cortex
PCL: Paracentral lobule
PMd: Dorsal premotor cortex
PMv: Ventral premotor cortex
preSMA: Pre-supplementary motor area
RBS-R: Repetitive behavior scale-revised
RD: Radial diffusivity
SRS-2: Social responsiveness scale, adult self-report
S1: Primary somatosensory cortex
SFG: Superior frontal gyrus
SMA: Supplementary motor area
SMG: Supramarginal gyrus
SOG: Superior occipital gyrus
SPL: Superior parietal lobule
STG: Superior temporal gyrus
TCATT: Transcallosal Tract Template
WASI-II: Wechsler Abbreviated Scales of Intelligence, Second Edition
