# Supplementary material for: Transcallosal white matter and cortical gray matter variations in autistic adults aged 30–73 years
Source: Mol Autism. 2025 Mar 6;16:16. doi: 10.1186/s13229-025-00652-6 (PMC11884179; doi:10.1186/s13229-025-00652-6)
Supplement: Supplementary file 1 — Supplementary Material 1. [file 13229_2025_652_MOESM1_ESM.docx]

# **Supplementary materials**

**Supplementary Figure 1**

**
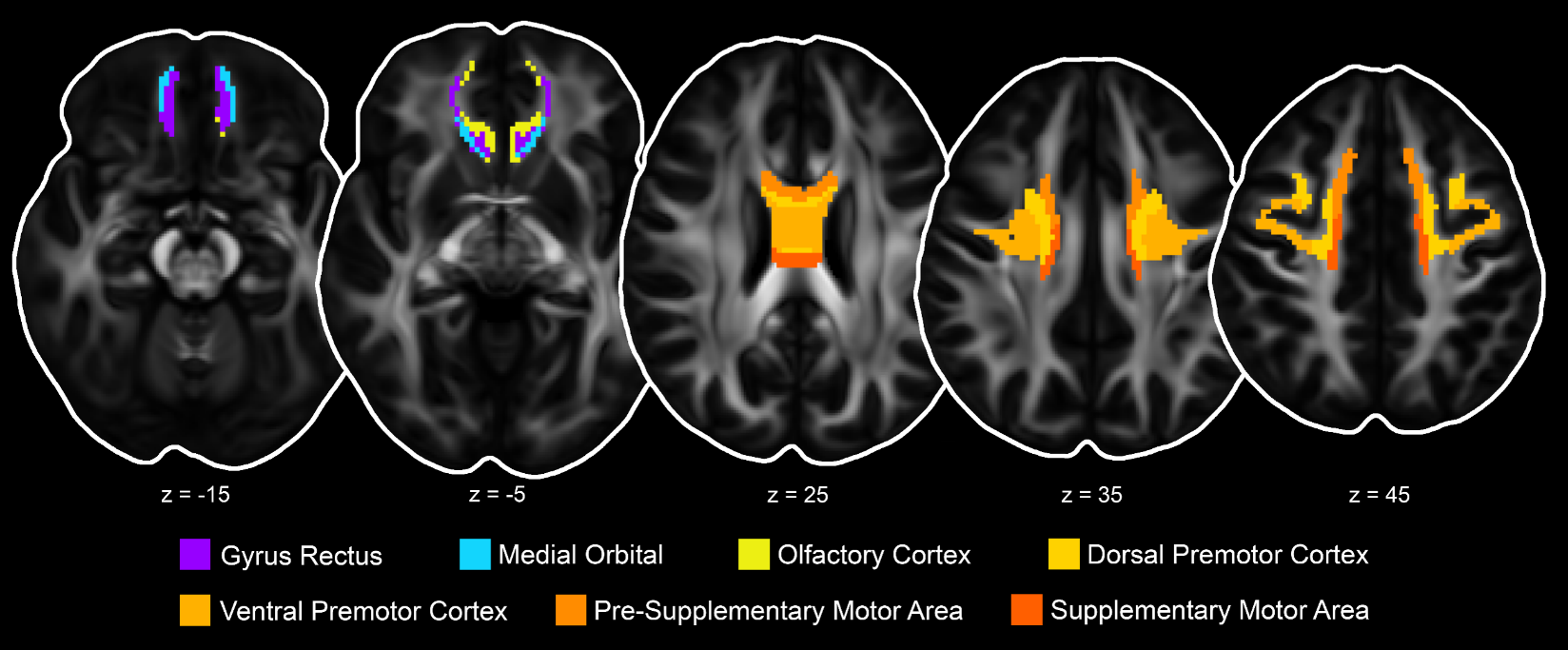
**

**Supplementary Figure 1.** Representation of the significant transcallosal white matter tracts including the gyrus rectus, medial orbital gyrus, olfactory cortex, dorsal premotor cortex, ventral premotor cortex, pre-supplementary motor area, and supplementary motor area in the axial view (z = -20, -15, -10, 0, 20).

**Supplementary Figure 2**


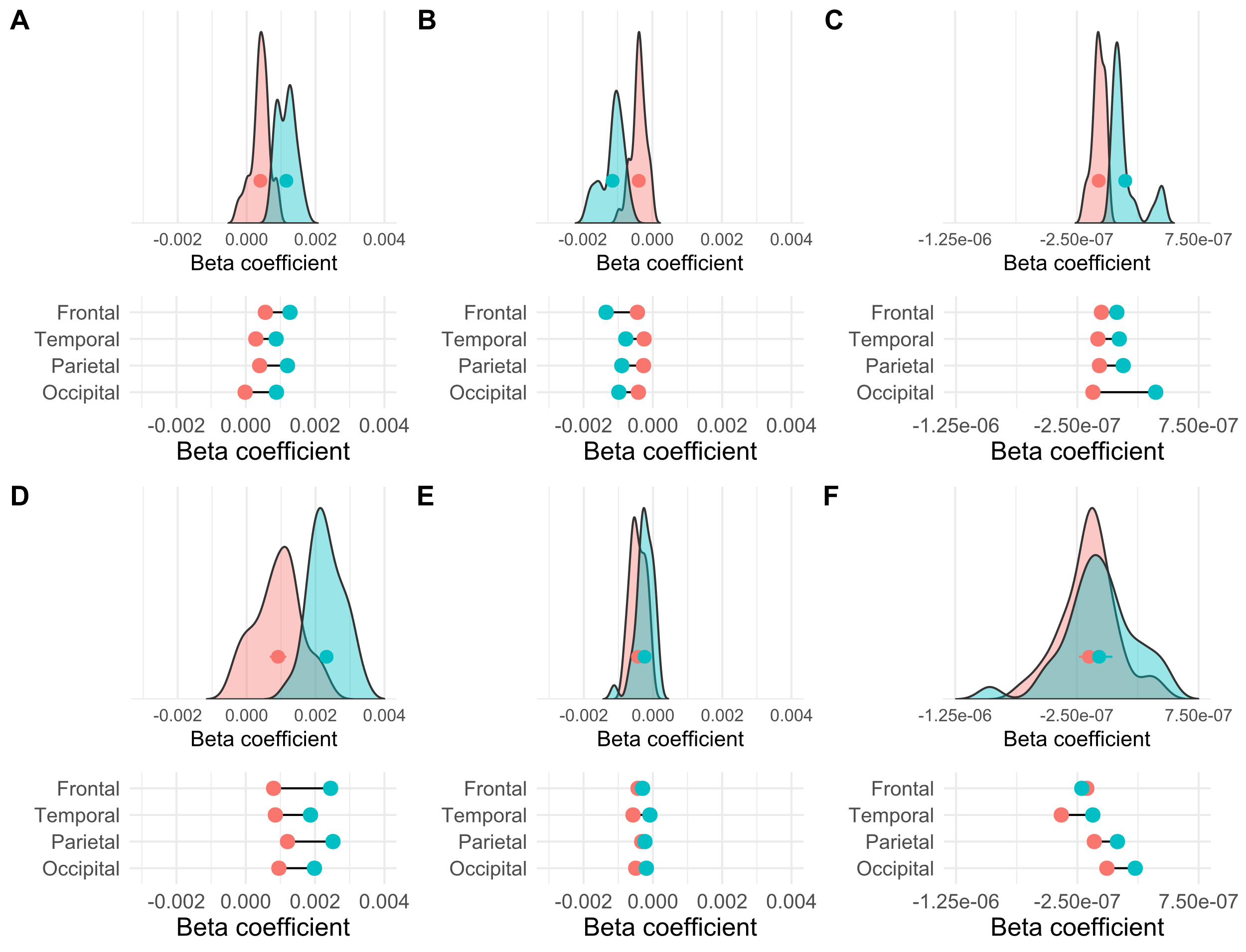


**FW**

**White matter**

**Gray matter**

**fwcFA**

**fwcMD**

**ASD**

**NT**

**Supplementary Figure 2.** Dispersion plots of $\beta$coefficient of 32 transcallosal tracts (top panel) and gray matter origin/endpoint ROIs (bottom panel) of autistic adults (rose red) and neurotypical controls (sky blue). Dispersion plots for free water, fwcFA, and fwcMD are shown from left to right. The
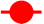
 that sits in the center of each dispersion plot represents the [Mean + SE] of $\beta$coefficient values for each group. The [Mean] of $\beta$coefficients derived from each of the frontal, temporal, parietal, and occipital regions are displayed at the bottom of each dispersion plot cluster and labeled by
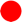
. The lines connecting these red and blue dots represent the $\beta$coefficient mean value difference between the autism and control groups.

**Supplementary Table Abbreviations**

AOG: anterior orbital gyrus, GR: gyrus rectus, IFG_oper: inferior frontal gyrus-pars opercularis, IFG_orb: inferior frontal gyrus-pars orbitalis, IFG_tri: inferior frontal gyrus-pars triangularis, LOG: lateral orbital gyrus, mFG: medial frontal gyrus, mOG: medial orbital gyrus, mOFG: medial orbitofrontal gyrus, MFG: middle frontal gyrus, OC: olfactory cortex, SFG: superior frontal gyrus, PMd: dorsal premotor cortex, PMv: ventral premotor cortex, preSMA: pre-supplementary motor area, M1: primary motor cortex, SMA: supplementary motor area, ITG: inferior temporal gyrus, MTG: middle temporal gyrus, STG: superior temporal gyrus, S1: primary somatosensory cortex, PCL: paracentral lobule, IPL: inferior parietal lobule, SPL: superior parietal lobule, SMG: supramarginal gyrus, ANG: angular gyrus, CAL: calcarine cortex, CUN: cuneus, LIG: lingual gyrus, IOG: inferior occipital gyrus, MOG: middle occipital gyrus, SOG: superior occipital gyrus.

**Supplementary Table 1.** Free water of 32 transcallosal tracts in autistic adults (ASD) and neurotypical controls (NT) and ANCOVA **results between the two groups**

|  | ASD | |  | NT | |  | η*_p_*^2^ |  | *p*_raw_ |  | *p*_FDR_ |
| --- | --- | --- | --- | --- | --- | --- | --- | --- | --- | --- | --- |
| ROIs | Mean | SD |  | Mean | SD |  |  |  |  |  |  |
| AOG | 0.213 | 0.0318 |  | 0.204 | 0.0291 |  | 0.036 |  | 0.086 |  | 0.172 |
| GR | 0.195 | 0.0322 |  | 0.180 | 0.0259 |  | 0.085 |  | **0.007** |  | **0.047** |
| IFG_oper | 0.177 | 0.0296 |  | 0.166 | 0.0335 |  | 0.059 |  | **0.027** |  | 0.103 |
| IFG_orb | 0.203 | 0.0291 |  | 0.195 | 0.0318 |  | 0.041 |  | 0.066 |  | 0.166 |
| IFG_tri | 0.169 | 0.0274 |  | 0.161 | 0.0325 |  | 0.038 |  | 0.074 |  | 0.168 |
| LOG | 0.209 | 0.0301 |  | 0.200 | 0.0297 |  | 0.047 |  | **0.047** |  | 0.152 |
| mFG | 0.176 | 0.0276 |  | 0.167 | 0.0337 |  | 0.039 |  | 0.071 |  | 0.166 |
| mOG | 0.224 | 0.0360 |  | 0.209 | 0.0300 |  | 0.079 |  | **0.009** |  | **0.047** |
| mOFG | 0.187 | 0.0285 |  | 0.175 | 0.0277 |  | 0.059 |  | **0.026** |  | 0.099 |
| MFG | 0.185 | 0.0277 |  | 0.177 | 0.0332 |  | 0.043 |  | 0.059 |  | 0.166 |
| OC | 0.188 | 0.0309 |  | 0.173 | 0.0261 |  | 0.088 |  | **0.006** |  | **0.047** |
| SFG | 0.171 | 0.0272 |  | 0.162 | 0.0340 |  | 0.035 |  | 0.090 |  | 0.173 |
| PMd | 0.167 | 0.0215 |  | 0.155 | 0.0272 |  | 0.098 |  | **0.004** |  | **0.047** |
| PMv | 0.176 | 0.0251 |  | 0.164 | 0.0293 |  | 0.089 |  | **0.006** |  | **0.047** |
| preSMA | 0.179 | 0.0245 |  | 0.167 | 0.0307 |  | 0.086 |  | **0.007** |  | **0.047** |
| M1 | 0.168 | 0.0201 |  | 0.161 | 0.0251 |  | 0.050 |  | **0.041** |  | 0.147 |
| SMA | 0.178 | 0.0222 |  | 0.166 | 0.0260 |  | 0.091 |  | **0.005** |  | **0.047** |
| ITG | 0.212 | 0.0249 |  | 0.206 | 0.0229 |  | 0.031 |  | 0.109 |  | 0.180 |
| MTG | 0.206 | 0.0259 |  | 0.200 | 0.0256 |  | 0.020 |  | 0.200 |  | 0.281 |
| STG | 0.190 | 0.0261 |  | 0.185 | 0.0272 |  | 0.019 |  | 0.207 |  | 0.281 |
| S1 | 0.195 | 0.0219 |  | 0.189 | 0.0274 |  | 0.033 |  | 0.099 |  | 0.176 |
| PCL | 0.176 | 0.0244 |  | 0.170 | 0.0261 |  | 0.031 |  | 0.109 |  | 0.180 |
| IPL | 0.179 | 0.0245 |  | 0.173 | 0.0288 |  | 0.026 |  | 0.142 |  | 0.217 |
| SPL | 0.189 | 0.0228 |  | 0.186 | 0.0312 |  | 0.009 |  | 0.404 |  | 0.480 |
| SMG | 0.184 | 0.0285 |  | 0.176 | 0.0296 |  | 0.036 |  | 0.082 |  | 0.169 |
| ANG | 0.183 | 0.0280 |  | 0.180 | 0.0338 |  | 0.009 |  | 0.402 |  | 0.480 |
| CAL | 0.231 | 0.0314 |  | 0.228 | 0.0324 |  | 0.003 |  | 0.627 |  | 0.683 |
| CUN | 0.190 | 0.0258 |  | 0.190 | 0.0279 |  | <0.001 |  | 0.853 |  | 0.857 |
| LIG | 0.257 | 0.0401 |  | 0.249 | 0.0397 |  | 0.014 |  | 0.276 |  | 0.359 |
| IOG | 0.230 | 0.0337 |  | 0.225 | 0.0350 |  | 0.008 |  | 0.414 |  | 0.480 |
| MOG | 0.192 | 0.0279 |  | 0.191 | 0.0306 |  | 0.003 |  | 0.636 |  | 0.683 |
| SOG | 0.203 | 0.0269 |  | 0.202 | 0.0289 |  | 0.002 |  | 0.691 |  | 0.719 |

**Supplementary Table 2.** Free water corrected fractional anisotropy (fwcFA) of 32 transcallosal tracts in autistic adults (ASD) and neurotypical controls (NT) and ANCOVA **results between the two groups**

|  | ASD | |  | NT | |  | η*_p_*^2^ |  | *p*_raw_ |  | *p*_FDR_ |
| --- | --- | --- | --- | --- | --- | --- | --- | --- | --- | --- | --- |
| ROIs | Mean | SD |  | Mean | SD |  |  |  |  |  |  |
| AOG | 0.628 | 0.0210 |  | 0.621 | 0.0313 |  | 0.006 |  | 0.497 |  | 0.924 |
| GR | 0.500 | 0.0192 |  | 0.495 | 0.0326 |  | <0.001 |  | 0.797 |  | 0.924 |
| IFG_oper | 0.525 | 0.0167 |  | 0.517 | 0.0234 |  | 0.026 |  | 0.144 |  | 0.924 |
| IFG_orb | 0.594 | 0.0136 |  | 0.591 | 0.0253 |  | 0.001 |  | 0.738 |  | 0.924 |
| IFG_tri | 0.475 | 0.0190 |  | 0.472 | 0.0228 |  | 0.001 |  | 0.756 |  | 0.924 |
| LOG | 0.634 | 0.0145 |  | 0.632 | 0.0264 |  | <0.001 |  | 0.985 |  | 0.984 |
| mFG | 0.562 | 0.0172 |  | 0.557 | 0.0291 |  | 0.002 |  | 0.707 |  | 0.924 |
| mOG | 0.591 | 0.0217 |  | 0.585 | 0.0325 |  | 0.004 |  | 0.554 |  | 0.924 |
| mOFG | 0.546 | 0.0193 |  | 0.542 | 0.0315 |  | <0.001 |  | 0.897 |  | 0.924 |
| MFG | 0.483 | 0.0160 |  | 0.480 | 0.0223 |  | <0.001 |  | 0.819 |  | 0.924 |
| OC | 0.447 | 0.0184 |  | 0.442 | 0.0309 |  | <0.001 |  | 0.799 |  | 0.924 |
| SFG | 0.534 | 0.0166 |  | 0.531 | 0.0281 |  | <0.001 |  | 0.822 |  | 0.924 |
| PMd | 0.504 | 0.0145 |  | 0.501 | 0.0232 |  | 0.002 |  | 0.664 |  | 0.924 |
| PMv | 0.504 | 0.0152 |  | 0.501 | 0.0218 |  | <0.001 |  | 0.831 |  | 0.924 |
| preSMA | 0.558 | 0.0178 |  | 0.557 | 0.0275 |  | 0.001 |  | 0.738 |  | 0.924 |
| M1 | 0.537 | 0.0164 |  | 0.533 | 0.0233 |  | 0.002 |  | 0.705 |  | 0.924 |
| SMA | 0.539 | 0.0181 |  | 0.535 | 0.0246 |  | 0.003 |  | 0.648 |  | 0.924 |
| ITG | 0.588 | 0.0154 |  | 0.586 | 0.0196 |  | 0.001 |  | 0.768 |  | 0.924 |
| MTG | 0.615 | 0.0145 |  | 0.610 | 0.0191 |  | 0.009 |  | 0.397 |  | 0.924 |
| STG | 0.549 | 0.0124 |  | 0.544 | 0.0210 |  | 0.014 |  | 0.275 |  | 0.924 |
| S1 | 0.557 | 0.0178 |  | 0.555 | 0.0225 |  | <0.001 |  | 0.866 |  | 0.924 |
| PCL | 0.518 | 0.0218 |  | 0.517 | 0.0237 |  | 0.001 |  | 0.769 |  | 0.924 |
| IPL | 0.533 | 0.0139 |  | 0.534 | 0.0188 |  | 0.010 |  | 0.366 |  | 0.924 |
| SPL | 0.584 | 0.0162 |  | 0.584 | 0.0190 |  | <0.001 |  | 0.886 |  | 0.924 |
| SMG | 0.539 | 0.0123 |  | 0.536 | 0.0175 |  | 0.002 |  | 0.694 |  | 0.924 |
| ANG | 0.549 | 0.0161 |  | 0.551 | 0.0222 |  | 0.009 |  | 0.379 |  | 0.924 |
| CAL | 0.597 | 0.0258 |  | 0.603 | 0.0287 |  | 0.027 |  | 0.135 |  | 0.924 |
| CUN | 0.628 | 0.0217 |  | 0.630 | 0.0250 |  | 0.007 |  | 0.463 |  | 0.924 |
| LIG | 0.583 | 0.0271 |  | 0.589 | 0.0289 |  | 0.019 |  | 0.206 |  | 0.924 |
| IOG | 0.653 | 0.0284 |  | 0.661 | 0.0281 |  | 0.036 |  | 0.085 |  | 0.924 |
| MOG | 0.605 | 0.0224 |  | 0.609 | 0.0244 |  | 0.019 |  | 0.208 |  | 0.924 |
| SOG | 0.641 | 0.0238 |  | 0.645 | 0.0255 |  | 0.019 |  | 0.216 |  | 0.924 |

**Supplementary Table 3.** Free water corrected mean diffusivity (fwcMD) of 32 transcallosal tracts in autistic adults (ASD) and neurotypical controls (NT) and ANCOVA **results between the two groups**

|  | ASD | |  | NT | |  | η*_p_*^2^ |  | *p*_raw_ |  | *p*_FDR_ |
| --- | --- | --- | --- | --- | --- | --- | --- | --- | --- | --- | --- |
| ROIs | Mean | SD |  | Mean | SD |  |  |  |  |  |  |
| AOG | 6.02E-04 | 2.19E-06 |  | 6.04E-04 | 1.29E-05 |  | 0.009 |  | 0.387 |  | 0.732 |
| GR | 5.99E-04 | 7.01E-06 |  | 6.01E-04 | 4.87E-06 |  | 0.027 |  | 0.134 |  | 0.647 |
| IFG_oper | 6.02E-04 | 1.63E-06 |  | 6.02E-04 | 4.59E-06 |  | 0.011 |  | 0.348 |  | 0.732 |
| IFG_orb | 6.03E-04 | 2.04E-06 |  | 6.04E-04 | 1.07E-05 |  | 0.007 |  | 0.436 |  | 0.732 |
| IFG_tri | 6.01E-04 | 8.86E-07 |  | 6.02E-04 | 4.52E-06 |  | 0.020 |  | 0.200 |  | 0.647 |
| LOG | 6.03E-04 | 2.81E-06 |  | 6.05E-04 | 1.30E-05 |  | 0.008 |  | 0.431 |  | 0.732 |
| mFG | 6.01E-04 | 8.80E-07 |  | 6.03E-04 | 9.74E-06 |  | 0.014 |  | 0.288 |  | 0.732 |
| mOG | 6.00E-04 | 6.87E-06 |  | 6.02E-04 | 6.46E-06 |  | 0.027 |  | 0.137 |  | 0.647 |
| mOFG | 6.01E-04 | 2.39E-06 |  | 6.03E-04 | 1.46E-05 |  | 0.015 |  | 0.264 |  | 0.724 |
| MFG | 6.01E-04 | 1.11E-06 |  | 6.02E-04 | 7.67E-06 |  | 0.016 |  | 0.248 |  | 0.692 |
| OC | 5.99E-04 | 7.27E-06 |  | 6.01E-04 | 4.33E-06 |  | 0.029 |  | 0.121 |  | 0.647 |
| SFG | 6.01E-04 | 8.23E-07 |  | 6.02E-04 | 8.23E-06 |  | 0.012 |  | 0.315 |  | 0.732 |
| PMd | 6.00E-04 | 3.04E-06 |  | 6.02E-04 | 3.55E-06 |  | 0.046 |  | **0.050** |  | 0.647 |
| PMv | 6.00E-04 | 6.04E-06 |  | 6.02E-04 | 4.34E-06 |  | 0.044 |  | 0.056 |  | 0.647 |
| preSMA | 6.02E-04 | 2.33E-06 |  | 6.03E-04 | 4.39E-06 |  | 0.010 |  | 0.362 |  | 0.732 |
| M1 | 6.02E-04 | 3.79E-06 |  | 6.04E-04 | 7.08E-06 |  | 0.033 |  | 0.100 |  | 0.647 |
| SMA | 6.02E-04 | 3.66E-06 |  | 6.03E-04 | 4.42E-06 |  | 0.028 |  | 0.128 |  | 0.647 |
| ITG | 6.00E-04 | 4.30E-06 |  | 6.02E-04 | 1.08E-05 |  | 0.017 |  | 0.240 |  | 0.724 |
| MTG | 6.02E-04 | 3.94E-06 |  | 6.03E-04 | 1.03E-05 |  | 0.009 |  | 0.382 |  | 0.732 |
| STG | 6.04E-04 | 3.60E-06 |  | 6.05E-04 | 7.41E-06 |  | 0.004 |  | 0.570 |  | 0.732 |
| S1 | 6.05E-04 | 5.21E-06 |  | 6.07E-04 | 9.79E-06 |  | 0.010 |  | 0.377 |  | 0.732 |
| PCL | 6.06E-04 | 4.86E-06 |  | 6.07E-04 | 7.50E-06 |  | 0.004 |  | 0.546 |  | 0.732 |
| IPL | 6.03E-04 | 3.59E-06 |  | 6.05E-04 | 1.09E-05 |  | 0.009 |  | 0.381 |  | 0.732 |
| SPL | 6.03E-04 | 3.50E-06 |  | 6.05E-04 | 1.52E-05 |  | 0.008 |  | 0.420 |  | 0.732 |
| SMG | 6.04E-04 | 4.18E-06 |  | 6.05E-04 | 7.76E-06 |  | 0.007 |  | 0.446 |  | 0.732 |
| ANG | 6.01E-04 | 3.11E-06 |  | 6.04E-04 | 1.86E-05 |  | 0.011 |  | 0.340 |  | 0.732 |
| CAL | 5.98E-04 | 4.77E-06 |  | 6.07E-04 | 4.81E-05 |  | 0.013 |  | 0.299 |  | 0.732 |
| CUN | 6.02E-04 | 3.34E-06 |  | 6.08E-04 | 4.31E-05 |  | 0.009 |  | 0.401 |  | 0.744 |
| LIG | 5.97E-04 | 7.72E-06 |  | 6.04E-04 | 3.09E-05 |  | 0.019 |  | 0.212 |  | 0.647 |
| IOG | 5.99E-04 | 5.64E-06 |  | 6.06E-04 | 4.67E-05 |  | 0.012 |  | 0.328 |  | 0.732 |
| MOG | 6.01E-04 | 3.04E-06 |  | 6.06E-04 | 3.68E-05 |  | 0.010 |  | 0.363 |  | 0.732 |
| SOG | 6.02E-04 | 3.47E-06 |  | 6.08E-04 | 4.59E-05 |  | 0.009 |  | 0.386 |  | 0.732 |

**Supplementary Table 4.** Free water (FW) of 32 gray matter origin/endpoint ROIs in autistic adults (ASD) and neurotypical controls (NT) and ANCOVA **results between the two groups**

|  | ASD | |  | NT | |  | η*_p_*^2^ |  | *p*_raw_ |  | *p*_FDR_ |
| --- | --- | --- | --- | --- | --- | --- | --- | --- | --- | --- | --- |
| ROIs | Mean | SD |  | Mean | SD |  |  |  |  |  |  |
| AOG | 0.170 | 0.0321 |  | 0.164 | 0.0523 |  | 0.009 |  | 0.395 |  | 0.535 |
| GR | 0.659 | 0.0552 |  | 0.636 | 0.0635 |  | 0.059 |  | **0.026** |  | 0.079 |
| IFG_oper | 0.334 | 0.0525 |  | 0.316 | 0.0580 |  | 0.063 |  | **0.021** |  | 0.079 |
| IFG_orb | 0.355 | 0.0619 |  | 0.338 | 0.0569 |  | 0.033 |  | 0.096 |  | 0.149 |
| IFG_tri | 0.305 | 0.0424 |  | 0.287 | 0.0506 |  | 0.062 |  | **0.022** |  | 0.079 |
| LOG | 0.423 | 0.0514 |  | 0.402 | 0.0612 |  | 0.046 |  | 0.051 |  | 0.090 |
| mFG | 0.316 | 0.0514 |  | 0.299 | 0.0571 |  | 0.052 |  | **0.036** |  | 0.084 |
| mOG | 0.436 | 0.0518 |  | 0.413 | 0.0515 |  | 0.068 |  | **0.016** |  | 0.079 |
| mOFG | 0.330 | 0.0487 |  | 0.306 | 0.0427 |  | 0.083 |  | **0.008** |  | 0.064 |
| MFG | 0.243 | 0.0390 |  | 0.236 | 0.0663 |  | 0.015 |  | 0.274 |  | 0.385 |
| OC | 0.520 | 0.0727 |  | 0.489 | 0.0680 |  | 0.068 |  | **0.016** |  | 0.079 |
| SFG | 0.307 | 0.0547 |  | 0.289 | 0.0564 |  | 0.055 |  | **0.031** |  | 0.079 |
| PMd | 0.549 | 0.0491 |  | 0.528 | 0.0459 |  | 0.101 |  | **0.003** |  | 0.054 |
| PMv | 0.305 | 0.0354 |  | 0.296 | 0.0410 |  | 0.038 |  | 0.074 |  | 0.120 |
| preSMA | 0.449 | 0.0617 |  | 0.425 | 0.0513 |  | 0.080 |  | **0.009** |  | 0.064 |
| M1 | 0.484 | 0.0420 |  | 0.471 | 0.0439 |  | 0.056 |  | **0.031** |  | 0.079 |
| SMA | 0.380 | 0.0584 |  | 0.363 | 0.0447 |  | 0.047 |  | **0.047** |  | 0.090 |
| ITG | 0.468 | 0.0648 |  | 0.442 | 0.0573 |  | 0.062 |  | **0.022** |  | 0.079 |
| MTG | 0.225 | 0.0311 |  | 0.217 | 0.0413 |  | 0.023 |  | 0.171 |  | 0.246 |
| STG | 0.311 | 0.0359 |  | 0.299 | 0.0412 |  | 0.056 |  | **0.030** |  | 0.079 |
| S1 | 0.435 | 0.0526 |  | 0.432 | 0.0525 |  | 0.011 |  | 0.337 |  | 0.421 |
| PCL | 0.325 | 0.0545 |  | 0.311 | 0.0500 |  | 0.037 |  | 0.078 |  | 0.129 |
| IPL | 0.210 | 0.0305 |  | 0.203 | 0.0508 |  | 0.017 |  | 0.236 |  | 0.327 |
| SPL | 0.308 | 0.0476 |  | 0.304 | 0.0703 |  | 0.007 |  | 0.462 |  | 0.540 |
| SMG | 0.261 | 0.0356 |  | 0.251 | 0.0477 |  | 0.042 |  | 0.062 |  | 0.114 |
| ANG | 0.223 | 0.0339 |  | 0.219 | 0.0606 |  | 0.005 |  | 0.517 |  | 0.577 |
| CAL | 0.299 | 0.0393 |  | 0.276 | 0.0428 |  | 0.119 |  | **0.001** |  | **0.045** |
| CUN | 0.295 | 0.0403 |  | 0.285 | 0.0426 |  | 0.028 |  | 0.130 |  | 0.189 |
| LIG | 0.395 | 0.0517 |  | 0.380 | 0.0456 |  | 0.050 |  | **0.040** |  | 0.090 |
| IOG | 0.533 | 0.0688 |  | 0.530 | 0.0602 |  | 0.005 |  | 0.515 |  | 0.553 |
| MOG | 0.239 | 0.0663 |  | 0.231 | 0.1018 |  | 0.004 |  | 0.590 |  | 0.621 |
| SOG | 0.244 | 0.0503 |  | 0.236 | 0.0752 |  | 0.007 |  | 0.463 |  | 0.540 |

**Supplementary Table 5.** Free water corrected fractional anisotropy (fwcFA) of 32 gray matter origin/endpoint ROIs in autistic adults (ASD) and neurotypical controls (NT) and ANCOVA **results between the two groups**

|  | ASD | |  | NT | |  | η*_p_*^2^ |  | *p*_raw_ |  | *p*_FDR_ |
| --- | --- | --- | --- | --- | --- | --- | --- | --- | --- | --- | --- |
| ROIs | Mean | SD |  | Mean | SD |  |  |  |  |  |  |
| AOG | 0.337 | 0.0184 |  | 0.341 | 0.0381 |  | 0.014 |  | 0.292 |  | 0.935 |
| GR | 0.155 | 0.0304 |  | 0.145 | 0.0304 |  | 0.023 |  | 0.165 |  | 0.935 |
| IFG_oper | 0.205 | 0.0180 |  | 0.203 | 0.0369 |  | <0.001 |  | 0.844 |  | 0.935 |
| IFG_orb | 0.190 | 0.0345 |  | 0.190 | 0.0326 |  | <0.001 |  | 0.807 |  | 0.935 |
| IFG_tri | 0.205 | 0.0231 |  | 0.204 | 0.0352 |  | <0.001 |  | 0.933 |  | 0.935 |
| LOG | 0.199 | 0.0280 |  | 0.194 | 0.0289 |  | 0.004 |  | 0.555 |  | 0.935 |
| mFG | 0.233 | 0.0116 |  | 0.231 | 0.0249 |  | 0.001 |  | 0.742 |  | 0.935 |
| mOG | 0.183 | 0.0266 |  | 0.177 | 0.0265 |  | 0.008 |  | 0.408 |  | 0.935 |
| mOFG | 0.204 | 0.0233 |  | 0.201 | 0.0359 |  | 0.001 |  | 0.757 |  | 0.935 |
| MFG | 0.251 | 0.0146 |  | 0.254 | 0.0392 |  | 0.004 |  | 0.586 |  | 0.935 |
| OC | 0.210 | 0.0424 |  | 0.202 | 0.0335 |  | 0.008 |  | 0.411 |  | 0.935 |
| SFG | 0.226 | 0.0118 |  | 0.224 | 0.0259 |  | 0.002 |  | 0.663 |  | 0.935 |
| PMd | 0.216 | 0.0144 |  | 0.211 | 0.0249 |  | 0.013 |  | 0.300 |  | 0.935 |
| PMv | 0.269 | 0.0144 |  | 0.267 | 0.0243 |  | 0.001 |  | 0.766 |  | 0.935 |
| preSMA | 0.229 | 0.0152 |  | 0.225 | 0.0274 |  | 0.007 |  | 0.457 |  | 0.935 |
| M1 | 0.243 | 0.0136 |  | 0.240 | 0.0248 |  | 0.008 |  | 0.430 |  | 0.935 |
| SMA | 0.250 | 0.0160 |  | 0.243 | 0.0199 |  | 0.026 |  | 0.141 |  | 0.935 |
| ITG | 0.173 | 0.0265 |  | 0.167 | 0.0398 |  | 0.005 |  | 0.533 |  | 0.935 |
| MTG | 0.187 | 0.0271 |  | 0.185 | 0.0344 |  | <0.001 |  | 0.935 |  | 0.935 |
| STG | 0.205 | 0.0213 |  | 0.203 | 0.0335 |  | <0.001 |  | 0.902 |  | 0.935 |
| S1 | 0.222 | 0.0160 |  | 0.220 | 0.0303 |  | 0.002 |  | 0.667 |  | 0.935 |
| PCL | 0.203 | 0.0131 |  | 0.202 | 0.0217 |  | <0.001 |  | 0.808 |  | 0.935 |
| IPL | 0.277 | 0.0152 |  | 0.282 | 0.0235 |  | 0.026 |  | 0.145 |  | 0.935 |
| SPL | 0.240 | 0.0137 |  | 0.241 | 0.0366 |  | <0.001 |  | 0.850 |  | 0.935 |
| SMG | 0.220 | 0.0123 |  | 0.222 | 0.0287 |  | 0.006 |  | 0.485 |  | 0.935 |
| ANG | 0.250 | 0.0163 |  | 0.250 | 0.0280 |  | 0.003 |  | 0.651 |  | 0.935 |
| CAL | 0.192 | 0.0213 |  | 0.193 | 0.0358 |  | <0.001 |  | 0.803 |  | 0.935 |
| CUN | 0.186 | 0.0251 |  | 0.182 | 0.0459 |  | 0.002 |  | 0.669 |  | 0.935 |
| LIG | 0.176 | 0.0211 |  | 0.174 | 0.0413 |  | <0.001 |  | 0.853 |  | 0.935 |
| IOG | 0.174 | 0.0279 |  | 0.163 | 0.0429 |  | 0.021 |  | 0.189 |  | 0.935 |
| MOG | 0.179 | 0.0244 |  | 0.180 | 0.0318 |  | 0.001 |  | 0.730 |  | 0.935 |
| SOG | 0.211 | 0.0244 |  | 0.207 | 0.0336 |  | 0.003 |  | 0.617 |  | 0.935 |

**Supplementary Table 6.** Free water corrected mean diffusivity (fwcMD) of 32 gray matter origin/endpoint ROIs in autistic adults (ASD) and neurotypical controls (NT) and ANCOVA **results between the two groups**

|  | ASD | |  | NT | |  | η*_p_*^2^ |  | *p*_raw_ |  | *p*_FDR_ |
| --- | --- | --- | --- | --- | --- | --- | --- | --- | --- | --- | --- |
| ROIs | Mean | SD |  | Mean | SD |  |  |  |  |  |  |
| AOG | 6.00E-04 | 9.72E-07 |  | 6.03E-04 | 2.27E-05 |  | 0.012 |  | 0.328 |  | 0.493 |
| GR | 4.82E-04 | 2.51E-05 |  | 4.89E-04 | 2.02E-05 |  | 0.032 |  | 0.106 |  | 0.176 |
| IFG_oper | 5.93E-04 | 9.56E-06 |  | 5.97E-04 | 1.50E-05 |  | 0.033 |  | 0.101 |  | 0.176 |
| IFG_orb | 5.82E-04 | 1.69E-05 |  | 5.85E-04 | 1.09E-05 |  | 0.021 |  | 0.189 |  | 0.222 |
| IFG_tri | 5.91E-04 | 7.28E-06 |  | 5.95E-04 | 1.39E-05 |  | 0.033 |  | 0.099 |  | 0.176 |
| LOG | 5.31E-04 | 1.80E-05 |  | 5.37E-04 | 1.46E-05 |  | 0.055 |  | **0.032** |  | 0.142 |
| mFG | 5.87E-04 | 1.41E-05 |  | 5.92E-04 | 1.19E-05 |  | 0.050 |  | **0.042** |  | 0.145 |
| mOG | 5.50E-04 | 1.30E-05 |  | 5.54E-04 | 1.25E-05 |  | 0.035 |  | 0.086 |  | 0.176 |
| mOFG | 5.87E-04 | 1.19E-05 |  | 5.93E-04 | 1.59E-05 |  | 0.042 |  | 0.061 |  | 0.152 |
| MFG | 5.99E-04 | 1.11E-06 |  | 6.02E-04 | 1.81E-05 |  | 0.015 |  | 0.273 |  | 0.295 |
| OC | 5.41E-04 | 3.38E-05 |  | 5.51E-04 | 2.74E-05 |  | 0.041 |  | 0.064 |  | 0.170 |
| SFG | 5.93E-04 | 8.76E-06 |  | 5.97E-04 | 7.87E-06 |  | 0.069 |  | **0.016** |  | 0.130 |
| PMd | 5.12E-04 | 2.10E-05 |  | 5.24E-04 | 1.52E-05 |  | 0.125 |  | **<0.001** |  | **0.006** |
| PMv | 5.76E-04 | 5.72E-06 |  | 5.78E-04 | 8.38E-06 |  | 0.025 |  | 0.152 |  | 0.204 |
| preSMA | 5.50E-04 | 2.36E-05 |  | 5.61E-04 | 1.36E-05 |  | 0.091 |  | **0.005** |  | 0.077 |
| M1 | 5.39E-04 | 1.33E-05 |  | 5.45E-04 | 1.38E-05 |  | 0.052 |  | **0.036** |  | 0.142 |
| SMA | 5.67E-04 | 1.78E-05 |  | 5.73E-04 | 1.22E-05 |  | 0.050 |  | **0.042** |  | 0.145 |
| ITG | 4.99E-04 | 2.49E-05 |  | 5.06E-04 | 2.57E-05 |  | 0.025 |  | 0.151 |  | 0.204 |
| MTG | 5.98E-04 | 4.68E-06 |  | 6.00E-04 | 8.73E-06 |  | 0.028 |  | 0.125 |  | 0.176 |
| STG | 5.83E-04 | 9.59E-06 |  | 5.88E-04 | 1.49E-05 |  | 0.051 |  | **0.039** |  | 0.142 |
| S1 | 5.47E-04 | 1.25E-05 |  | 5.52E-04 | 1.37E-05 |  | 0.036 |  | 0.085 |  | 0.176 |
| PCL | 5.90E-04 | 1.37E-05 |  | 5.94E-04 | 9.60E-06 |  | 0.026 |  | 0.139 |  | 0.204 |
| IPL | 5.99E-04 | 4.14E-06 |  | 6.02E-04 | 1.31E-05 |  | 0.020 |  | 0.204 |  | 0.222 |
| SPL | 5.94E-04 | 6.08E-06 |  | 6.02E-04 | 3.52E-05 |  | 0.021 |  | 0.186 |  | 0.176 |
| SMG | 5.98E-04 | 3.49E-06 |  | 6.00E-04 | 9.39E-06 |  | 0.031 |  | 0.107 |  | 0.142 |
| ANG | 5.98E-04 | 4.51E-06 |  | 6.04E-04 | 2.83E-05 |  | 0.016 |  | 0.252 |  | 0.269 |
| CAL | 5.96E-04 | 4.32E-06 |  | 5.99E-04 | 1.02E-05 |  | 0.040 |  | 0.067 |  | 0.142 |
| CUN | 5.86E-04 | 9.82E-06 |  | 5.94E-04 | 4.26E-05 |  | 0.015 |  | 0.272 |  | 0.334 |
| LIG | 5.73E-04 | 1.73E-05 |  | 5.77E-04 | 1.71E-05 |  | 0.014 |  | 0.276 |  | 0.328 |
| IOG | 4.94E-04 | 2.23E-05 |  | 4.97E-04 | 4.37E-05 |  | 0.003 |  | 0.641 |  | 0.722 |
| MOG | 5.93E-04 | 2.12E-05 |  | 6.03E-04 | 3.86E-05 |  | 0.025 |  | 0.154 |  | 0.204 |
| SOG | 5.93E-04 | 1.17E-05 |  | 6.04E-04 | 4.80E-05 |  | 0.020 |  | 0.205 |  | 0.204 |

**Supplementary Table 7.** Nonparametric partial correlation results between dMRI measures (free water, fwcFA, and fwcMD) across 32 transcallosal tracts and age in autistic adults (ASD) and neurotypical controls (NT)

|  | Free water | | | | |  | fwcFA | | | | |  | fwcMD | | | | |
| --- | --- | --- | --- | --- | --- | --- | --- | --- | --- | --- | --- | --- | --- | --- | --- | --- | --- |
|  | ASD | |  | NT | |  | ASD | |  | NT | |  | ASD | |  | NT | |
| ROI | R | *p*_FDR_ |  | R | *p*_FDR_ |  | R | *p*_FDR_ |  | R | *p*_FDR_ |  | R | *p*_FDR_ |  | R | *p*_FDR_ |
| AOG | 0.224 | 0.475 |  | 0.508 | **<0.001** |  | -0.201 | 0.368 |  | -0.620 | **0.001** |  | -0.056 | 0.776 |  | 0.103 | 0.853 |
| GR | 0.190 | 0.547 |  | 0.382 | **0.008** |  | -0.406 | 0.098 |  | -0.681 | **<0.001** |  | -0.168 | 0.521 |  | 0.119 | 0.835 |
| IFG_oper | 0.313 | 0.475 |  | 0.541 | **<0.001** |  | -0.455 | 0.058 |  | -0.606 | **0.002** |  | -0.116 | 0.677 |  | 0.226 | 0.624 |
| IFG_orb | 0.151 | 0.547 |  | 0.631 | **<0.001** |  | -0.156 | 0.374 |  | -0.548 | **0.002** |  | 0.007 | 0.970 |  | 0.090 | 0.853 |
| IFG_tri | 0.214 | 0.477 |  | 0.510 | **<0.001** |  | -0.536 | 0.058 |  | -0.581 | **0.002** |  | -0.088 | 0.722 |  | 0.197 | 0.624 |
| LOG | 0.210 | 0.475 |  | 0.610 | **<0.001** |  | -0.046 | 0.831 |  | -0.577 | **0.002** |  | -0.074 | 0.753 |  | 0.086 | 0.853 |
| mFG | 0.170 | 0.547 |  | 0.452 | **<0.001** |  | -0.258 | 0.230 |  | -0.638 | **0.001** |  | -0.108 | 0.697 |  | 0.132 | 0.835 |
| mOG | 0.266 | 0.475 |  | 0.536 | **<0.001** |  | -0.230 | 0.289 |  | -0.677 | **<0.001** |  | -0.096 | 0.753 |  | 0.121 | 0.835 |
| mOFG | 0.151 | 0.547 |  | 0.370 | **0.009** |  | -0.338 | 0.123 |  | -0.661 | **<0.001** |  | -0.227 | 0.492 |  | 0.119 | 0.835 |
| MFG | 0.217 | 0.475 |  | 0.545 | **<0.001** |  | -0.308 | 0.166 |  | -0.613 | **0.001** |  | -0.167 | 0.554 |  | 0.126 | 0.835 |
| OC | 0.170 | 0.547 |  | 0.334 | **0.014** |  | -0.405 | 0.123 |  | -0.669 | **<0.001** |  | -0.202 | 0.492 |  | 0.116 | 0.835 |
| SFG | 0.158 | 0.547 |  | 0.447 | **0.001** |  | -0.247 | 0.240 |  | -0.642 | **0.001** |  | -0.128 | 0.677 |  | 0.141 | 0.835 |
| PMd | 0.329 | 0.475 |  | 0.534 | **<0.001** |  | -0.193 | 0.313 |  | -0.548 | **0.002** |  | -0.296 | 0.492 |  | 0.133 | 0.835 |
| PMv | 0.384 | 0.475 |  | 0.526 | **<0.001** |  | -0.174 | 0.374 |  | -0.447 | **0.007** |  | -0.346 | 0.492 |  | 0.152 | 0.835 |
| preSMA | 0.291 | 0.475 |  | 0.552 | **<0.001** |  | -0.228 | 0.240 |  | -0.635 | **<0.001** |  | -0.067 | 0.753 |  | 0.183 | 0.805 |
| M1 | 0.205 | 0.475 |  | 0.481 | **0.002** |  | -0.224 | 0.313 |  | -0.536 | **0.001** |  | -0.252 | 0.492 |  | 0.105 | 0.835 |
| SMA | 0.273 | 0.475 |  | 0.547 | **<0.001** |  | -0.237 | 0.240 |  | -0.552 | **0.002** |  | -0.147 | 0.677 |  | 0.096 | 0.835 |
| ITG | 0.129 | 0.611 |  | 0.384 | **0.012** |  | -0.253 | 0.240 |  | -0.500 | **0.006** |  | -0.280 | 0.492 |  | 0.078 | 0.853 |
| MTG | 0.144 | 0.547 |  | 0.405 | **0.008** |  | -0.160 | 0.368 |  | -0.528 | **0.002** |  | -0.237 | 0.655 |  | 0.123 | 0.835 |
| STG | 0.091 | 0.676 |  | 0.427 | **0.004** |  | -0.160 | 0.368 |  | -0.403 | **0.015** |  | -0.132 | 0.701 |  | 0.185 | 0.805 |
| S1 | 0.246 | 0.475 |  | 0.553 | **<0.001** |  | -0.038 | 0.880 |  | -0.543 | **0.001** |  | -0.146 | 0.677 |  | 0.137 | 0.835 |
| PCL | 0.189 | 0.475 |  | 0.518 | **<0.001** |  | -0.351 | 0.123 |  | -0.544 | **<0.001** |  | -0.105 | 0.701 |  | 0.086 | 0.851 |
| IPL | 0.158 | 0.547 |  | 0.477 | **0.002** |  | -0.198 | 0.282 |  | -0.573 | **<0.001** |  | -0.174 | 0.677 |  | 0.146 | 0.835 |
| SPL | 0.114 | 0.611 |  | 0.451 | **0.002** |  | -0.064 | 0.767 |  | -0.388 | **0.012** |  | -0.210 | 0.677 |  | 0.136 | 0.835 |
| SMG | 0.196 | 0.475 |  | 0.462 | **0.002** |  | -0.010 | 0.945 |  | -0.579 | **0.001** |  | -0.201 | 0.677 |  | 0.146 | 0.835 |
| ANG | 0.148 | 0.547 |  | 0.438 | **0.004** |  | -0.320 | 0.096 |  | -0.487 | **0.004** |  | -0.292 | 0.492 |  | 0.134 | 0.835 |
| CAL | 0.018 | 0.944 |  | 0.305 | **0.037** |  | -0.212 | 0.289 |  | -0.407 | **0.016** |  | -0.281 | 0.492 |  | 0.112 | 0.851 |
| CUN | -0.111 | 0.676 |  | 0.369 | **0.011** |  | -0.198 | 0.295 |  | -0.404 | **0.016** |  | -0.268 | 0.492 |  | 0.122 | 0.835 |
| LIG | 0.060 | 0.775 |  | 0.273 | 0.065 |  | -0.162 | 0.368 |  | -0.403 | **0.021** |  | -0.247 | 0.492 |  | 0.090 | 0.853 |
| IOG | 0.016 | 0.944 |  | 0.289 | **0.047** |  | -0.190 | 0.336 |  | -0.434 | **0.012** |  | -0.303 | 0.492 |  | 0.104 | 0.853 |
| MOG | -0.009 | 0.958 |  | 0.361 | **0.014** |  | -0.168 | 0.368 |  | -0.517 | **0.004** |  | -0.303 | 0.492 |  | 0.118 | 0.835 |
| SOG | -0.059 | 0.817 |  | 0.362 | **0.012** |  | -0.143 | 0.406 |  | -0.470 | **0.006** |  | -0.310 | 0.492 |  | 0.118 | 0.835 |

**Supplementary Table 8.** Nonparametric partial correlation results between diffusion measures (free water, fwcFA, and fwcMD) in across 32 gray matter origin/endpoint ROIs and age in autistic adults (ASD) and neurotypical controls (NT)

|  | Free water | | | | |  | fwcFA | | | | |  | fwcMD | | | | |
| --- | --- | --- | --- | --- | --- | --- | --- | --- | --- | --- | --- | --- | --- | --- | --- | --- | --- |
|  | ASD | |  | NT | |  | ASD | |  | NT | |  | ASD | |  | NT | |
| ROI | R | *p*_FDR_ |  | R | *p*_FDR_ |  | R | *p*_FDR_ |  | R | *p*_FDR_ |  | R | *p*_FDR_ |  | R | *p*_FDR_ |
| AOG | -0.078 | 0.856 |  | 0.268 | **0.013** |  | -0.349 | 0.454 |  | -0.355 | 0.282 |  | 0.047 | 0.871 |  | 0.128 | 0.930 |
| GR | 0.064 | 0.856 |  | 0.499 | **0.002** |  | -0.165 | 0.604 |  | -0.012 | 0.993 |  | -0.060 | 0.839 |  | -0.268 | 0.344 |
| IFG_oper | 0.262 | 0.207 |  | 0.687 | **<0.001** |  | -0.252 | 0.604 |  | -0.108 | 0.993 |  | -0.164 | 0.611 |  | -0.108 | 0.972 |
| IFG_orb | -0.059 | 0.856 |  | 0.504 | **0.001** |  | -0.213 | 0.604 |  | -0.166 | 0.993 |  | -0.028 | 0.899 |  | -0.326 | 0.198 |
| IFG_tri | 0.206 | 0.373 |  | 0.516 | **<0.001** |  | -0.394 | 0.454 |  | -0.121 | 0.993 |  | -0.292 | 0.269 |  | -0.068 | 0.972 |
| LOG | 0.017 | 0.963 |  | 0.413 | **0.002** |  | -0.289 | 0.604 |  | -0.256 | 0.467 |  | -0.058 | 0.839 |  | -0.436 | 0.090 |
| mFG | 0.279 | 0.207 |  | 0.605 | **<0.001** |  | -0.219 | 0.604 |  | -0.143 | 0.993 |  | -0.230 | 0.435 |  | -0.419 | 0.198 |
| mOG | 0.102 | 0.783 |  | 0.496 | **0.003** |  | -0.224 | 0.604 |  | -0.102 | 0.993 |  | -0.150 | 0.692 |  | -0.178 | 0.848 |
| mOFG | 0.032 | 0.948 |  | 0.504 | **0.002** |  | -0.253 | 0.604 |  | -0.076 | 0.993 |  | -0.147 | 0.657 |  | 0.024 | 0.973 |
| MFG | 0.239 | 0.330 |  | 0.480 | **<0.001** |  | -0.199 | 0.604 |  | -0.064 | 0.993 |  | -0.095 | 0.832 |  | 0.114 | 0.972 |
| OC | 0.180 | 0.450 |  | 0.423 | **0.007** |  | -0.204 | 0.604 |  | -0.100 | 0.993 |  | -0.172 | 0.641 |  | -0.412 | 0.192 |
| SFG | 0.270 | 0.207 |  | 0.654 | **<0.001** |  | -0.100 | 0.728 |  | -0.058 | 0.993 |  | -0.190 | 0.553 |  | -0.239 | 0.531 |
| PMd | 0.414 | 0.075 |  | 0.692 | **<0.001** |  | -0.166 | 0.607 |  | -0.016 | 0.993 |  | -0.182 | 0.589 |  | -0.219 | 0.531 |
| PMv | 0.303 | 0.207 |  | 0.625 | **<0.001** |  | -0.278 | 0.604 |  | -0.263 | 0.467 |  | -0.192 | 0.553 |  | -0.021 | 0.973 |
| preSMA | 0.229 | 0.324 |  | 0.671 | **<0.001** |  | -0.094 | 0.716 |  | -0.065 | 0.993 |  | -0.142 | 0.641 |  | -0.150 | 0.848 |
| M1 | 0.317 | 0.207 |  | 0.641 | **<0.001** |  | -0.038 | 0.843 |  | 0.041 | 0.993 |  | -0.057 | 0.839 |  | -0.057 | 0.973 |
| SMA | 0.179 | 0.454 |  | 0.573 | **<0.001** |  | -0.153 | 0.604 |  | -0.088 | 0.993 |  | -0.089 | 0.832 |  | -0.271 | 0.402 |
| ITG | 0.199 | 0.324 |  | 0.367 | **0.006** |  | -0.234 | 0.604 |  | 0.022 | 0.993 |  | -0.299 | 0.209 |  | -0.068 | 0.972 |
| MTG | 0.160 | 0.459 |  | 0.441 | **<0.001** |  | -0.281 | 0.604 |  | -0.106 | 0.993 |  | -0.337 | 0.209 |  | 0.060 | 0.973 |
| STG | 0.270 | 0.207 |  | 0.646 | **<0.001** |  | -0.225 | 0.604 |  | -0.009 | 0.993 |  | -0.351 | 0.209 |  | -0.207 | 0.531 |
| S1 | 0.457 | 0.075 |  | 0.686 | **<0.001** |  | -0.107 | 0.716 |  | 0.050 | 0.993 |  | -0.326 | 0.209 |  | -0.109 | 0.972 |
| PCL | 0.246 | 0.207 |  | 0.606 | **<0.001** |  | -0.183 | 0.604 |  | -0.119 | 0.993 |  | -0.153 | 0.641 |  | -0.139 | 0.930 |
| IPL | 0.295 | 0.207 |  | 0.443 | **<0.001** |  | -0.389 | 0.454 |  | -0.341 | 0.282 |  | 0.193 | 0.839 |  | 0.123 | 0.972 |
| SPL | 0.164 | 0.450 |  | 0.520 | **<0.001** |  | -0.081 | 0.716 |  | 0.035 | 0.993 |  | -0.127 | 0.666 |  | 0.087 | 0.973 |
| SMG | 0.438 | 0.075 |  | 0.642 | **<0.001** |  | -0.265 | 0.604 |  | -0.123 | 0.993 |  | -0.467 | 0.209 |  | 0.020 | 0.973 |
| ANG | 0.226 | 0.373 |  | 0.390 | **0.001** |  | -0.384 | 0.454 |  | -0.175 | 0.993 |  | 0.143 | 0.839 |  | 0.129 | 0.930 |
| CAL | 0.348 | 0.162 |  | 0.533 | **0.002** |  | -0.194 | 0.604 |  | -0.093 | 0.993 |  | 0.025 | 0.871 |  | 0.051 | 0.973 |
| CUN | 0.172 | 0.469 |  | 0.524 | **<0.001** |  | -0.154 | 0.716 |  | -0.011 | 0.993 |  | 0.161 | 0.743 |  | 0.113 | 0.972 |
| LIG | 0.396 | 0.147 |  | 0.461 | **0.005** |  | -0.245 | 0.604 |  | -0.015 | 0.993 |  | -0.253 | 0.442 |  | 0.030 | 0.973 |
| IOG | 0.327 | 0.107 |  | 0.426 | **0.007** |  | -0.209 | 0.604 |  | 0.004 | 0.993 |  | -0.248 | 0.360 |  | -0.014 | 0.973 |
| MOG | -0.034 | 0.948 |  | 0.264 | **0.009** |  | -0.294 | 0.604 |  | -0.129 | 0.993 |  | 0.207 | 0.728 |  | 0.132 | 0.930 |
| SOG | <0.001 | 0.999 |  | 0.316 | **0.005** |  | -0.255 | 0.604 |  | -0.143 | 0.993 |  | 0.301 | 0.589 |  | 0.123 | 0.972 |

**Supplementary Table 9.** Quadratic regression results of diffusion measures (free water, fwcFA, and fwcMD) across 32 transcallosal tracts with age and age^2^ in autistic adults (ASD) and neurotypical controls (NT). Table shows FDR corrected p-values for age and age^2^

|  | Free water | | | | |  | fwcFA | | | | |  | fwcMD | | | | |
| --- | --- | --- | --- | --- | --- | --- | --- | --- | --- | --- | --- | --- | --- | --- | --- | --- | --- |
|  | ASD | |  | NT | |  | ASD | |  | NT | |  | ASD | |  | NT | |
| ROI | Age | Age^2^ |  | Age | Age^2^ |  | Age | Age^2^ |  | Age | Age^2^ |  | Age | Age^2^ |  | Age | Age^2^ |
| AOG | 0.611 | 0.707 |  | 0.888 | 0.639 |  | 0.446 | 0.540 |  | 0.601 | 0.857 |  | 0.693 | 0.820 |  | 0.842 | 0.901 |
| GR | 0.611 | 0.707 |  | 0.888 | 0.639 |  | 0.320 | 0.426 |  | 0.564 | 0.857 |  | 0.574 | 0.660 |  | 0.842 | 0.901 |
| IFG_oper | 0.636 | 0.790 |  | 0.888 | 0.639 |  | 0.214 | 0.348 |  | 0.397 | 0.834 |  | 0.491 | 0.636 |  | 0.842 | 0.901 |
| IFG_orb | 0.611 | 0.707 |  | 0.888 | 0.639 |  | 0.231 | 0.348 |  | 0.470 | 0.834 |  | 0.491 | 0.636 |  | 0.865 | 0.915 |
| IFG_tri | 0.684 | 0.790 |  | 0.888 | 0.639 |  | **0.009** | **0.039** |  | 0.397 | 0.834 |  | 0.743 | 0.822 |  | 0.842 | 0.901 |
| LOG | 0.611 | 0.707 |  | 0.888 | 0.639 |  | 0.372 | 0.426 |  | 0.397 | 0.834 |  | 0.574 | 0.636 |  | 0.849 | 0.901 |
| mFG | 0.684 | 0.790 |  | 0.888 | 0.639 |  | 0.231 | 0.348 |  | 0.475 | 0.857 |  | 0.491 | 0.636 |  | 0.842 | 0.901 |
| mOG | 0.611 | 0.707 |  | 0.888 | 0.639 |  | 0.383 | 0.483 |  | 0.435 | 0.834 |  | 0.693 | 0.822 |  | 0.842 | 0.901 |
| mOFG | 0.611 | 0.707 |  | 0.888 | 0.639 |  | 0.231 | 0.361 |  | 0.601 | 0.857 |  | 0.491 | 0.636 |  | 0.842 | 0.901 |
| MFG | 0.611 | 0.707 |  | 0.888 | 0.639 |  | **0.037** | 0.070 |  | 0.397 | 0.834 |  | 0.574 | 0.675 |  | 0.842 | 0.901 |
| OC | 0.636 | 0.780 |  | 0.888 | 0.639 |  | 0.223 | 0.348 |  | 0.601 | 0.857 |  | 0.574 | 0.660 |  | 0.842 | 0.901 |
| SFG | 0.700 | 0.790 |  | 0.888 | 0.639 |  | 0.320 | 0.426 |  | 0.483 | 0.857 |  | 0.972 | 0.921 |  | 0.842 | 0.901 |
| PMd | 0.611 | 0.725 |  | 0.888 | 0.639 |  | 0.165 | 0.218 |  | 0.966 | 0.857 |  | 0.693 | 0.822 |  | 0.938 | 0.915 |
| PMv | 0.611 | 0.707 |  | 0.888 | 0.639 |  | 0.073 | 0.093 |  | 0.854 | 0.857 |  | 0.693 | 0.834 |  | 0.909 | 0.915 |
| preSMA | 0.636 | 0.790 |  | 0.888 | 0.639 |  | 0.372 | 0.466 |  | 0.601 | 0.857 |  | 0.574 | 0.636 |  | 0.842 | 0.901 |
| M1 | 0.611 | 0.707 |  | 0.888 | 0.639 |  | 0.065 | 0.093 |  | 0.966 | 0.857 |  | 0.794 | 0.921 |  | 0.970 | 0.981 |
| SMA | 0.930 | 0.902 |  | 0.888 | 0.639 |  | 0.320 | 0.426 |  | 0.991 | 0.857 |  | 0.693 | 0.822 |  | 0.842 | 0.901 |
| ITG | 0.611 | 0.707 |  | 0.888 | 0.639 |  | 0.174 | 0.256 |  | 0.601 | 0.857 |  | 0.491 | 0.636 |  | 0.849 | 0.901 |
| MTG | 0.611 | 0.707 |  | 0.888 | 0.639 |  | 0.172 | 0.218 |  | 0.601 | 0.857 |  | 0.491 | 0.636 |  | 0.842 | 0.901 |
| STG | 0.611 | 0.707 |  | 0.888 | 0.639 |  | 0.320 | 0.426 |  | 0.910 | 0.933 |  | 0.491 | 0.636 |  | 0.871 | 0.915 |
| S1 | 0.611 | 0.707 |  | 0.888 | 0.639 |  | 0.090 | 0.093 |  | 0.966 | 0.857 |  | 0.693 | 0.822 |  | 0.901 | 0.915 |
| PCL | 0.611 | 0.707 |  | 0.888 | 0.639 |  | **0.006** | **0.015** |  | 0.966 | 0.857 |  | 0.972 | 0.965 |  | 0.970 | 0.954 |
| IPL | 0.611 | 0.707 |  | 0.888 | 0.639 |  | 0.099 | 0.139 |  | 0.985 | 0.857 |  | 0.491 | 0.636 |  | 0.842 | 0.901 |
| SPL | 0.611 | 0.707 |  | 0.899 | 0.639 |  | 0.144 | 0.156 |  | 0.966 | 0.952 |  | 0.491 | 0.636 |  | 0.842 | 0.901 |
| SMG | 0.611 | 0.707 |  | 0.901 | 0.639 |  | 0.742 | 0.746 |  | 0.966 | 0.857 |  | 0.491 | 0.636 |  | 0.865 | 0.915 |
| ANG | 0.611 | 0.707 |  | 0.888 | 0.639 |  | 0.223 | 0.348 |  | 0.601 | 0.834 |  | 0.491 | 0.636 |  | 0.842 | 0.901 |
| CAL | 0.611 | 0.707 |  | 0.888 | 0.639 |  | 0.320 | 0.426 |  | 0.483 | 0.834 |  | 0.491 | 0.636 |  | 0.842 | 0.901 |
| CUN | 0.636 | 0.707 |  | 0.888 | 0.639 |  | 0.362 | 0.426 |  | 0.601 | 0.857 |  | 0.491 | 0.636 |  | 0.842 | 0.901 |
| LIG | 0.611 | 0.707 |  | 0.888 | 0.639 |  | 0.412 | 0.483 |  | 0.475 | 0.834 |  | 0.491 | 0.636 |  | 0.842 | 0.901 |
| IOG | 0.611 | 0.707 |  | 0.888 | 0.639 |  | 0.366 | 0.432 |  | 0.397 | 0.834 |  | 0.491 | 0.636 |  | 0.842 | 0.901 |
| MOG | 0.611 | 0.707 |  | 0.888 | 0.639 |  | 0.362 | 0.426 |  | 0.397 | 0.834 |  | 0.491 | 0.636 |  | 0.842 | 0.901 |
| SOG | 0.611 | 0.707 |  | 0.888 | 0.639 |  | 0.362 | 0.426 |  | 0.397 | 0.834 |  | 0.491 | 0.636 |  | 0.842 | 0.901 |

**Supplementary Table 10.** Quadratic regression results of diffusion measures (free water, fwcFA, and fwcMD) across 32 gray matter origin/endpoint ROIs with age and age^2^ in autistic adults (ASD) and neurotypical controls (NT). Table shows FDR corrected p-values for age and age^2^

|  | Free water | | | | |  | fwcFA | | | | |  | fwcMD | | | | |
| --- | --- | --- | --- | --- | --- | --- | --- | --- | --- | --- | --- | --- | --- | --- | --- | --- | --- |
|  | ASD | |  | NT | |  | ASD | |  | NT | |  | ASD | |  | NT | |
| ROI | Age | Age^2^ |  | Age | Age^2^ |  | Age | Age^2^ |  | Age | Age^2^ |  | Age | Age^2^ |  | Age | Age^2^ |
| AOG | 0.995 | 0.995 |  | 0.998 | 0.994 |  | 0.275 | 0.367 |  | 0.568 | 0.684 |  | 0.859 | 0.936 |  | 0.956 | 0.926 |
| GR | 0.995 | 0.995 |  | 0.998 | 0.994 |  | 0.702 | 0.794 |  | 0.568 | 0.564 |  | 0.778 | 0.765 |  | 0.999 | 0.974 |
| IFG_oper | 0.995 | 0.995 |  | 0.998 | 0.994 |  | 0.275 | 0.367 |  | 0.568 | 0.564 |  | 0.778 | 0.765 |  | 0.956 | 0.926 |
| IFG_orb | 0.995 | 0.995 |  | 0.998 | 0.994 |  | 0.313 | 0.399 |  | 0.568 | 0.564 |  | 0.710 | 0.765 |  | 0.956 | 0.926 |
| IFG_tri | 0.995 | 0.995 |  | 0.998 | 0.994 |  | 0.275 | 0.367 |  | 0.568 | 0.564 |  | 0.710 | 0.765 |  | 0.983 | 0.974 |
| LOG | 0.995 | 0.995 |  | 0.998 | 0.994 |  | 0.313 | 0.393 |  | 0.568 | 0.564 |  | 0.778 | 0.765 |  | 0.999 | 0.974 |
| mFG | 0.995 | 0.995 |  | 0.998 | 0.994 |  | 0.294 | 0.367 |  | 0.568 | 0.564 |  | 0.804 | 0.918 |  | 0.956 | 0.926 |
| mOG | 0.995 | 0.995 |  | 0.998 | 0.994 |  | 0.469 | 0.578 |  | 0.568 | 0.564 |  | 0.804 | 0.765 |  | 0.983 | 0.926 |
| mOFG | 0.995 | 0.995 |  | 0.998 | 0.994 |  | 0.313 | 0.393 |  | 0.568 | 0.564 |  | 0.778 | 0.765 |  | 0.956 | 0.926 |
| MFG | 0.995 | 0.995 |  | 0.998 | 0.994 |  | 0.116 | 0.172 |  | 0.793 | 0.784 |  | 0.859 | 0.938 |  | 0.956 | 0.926 |
| OC | 0.995 | 0.995 |  | 0.998 | 0.994 |  | 0.313 | 0.393 |  | 0.568 | 0.564 |  | 0.710 | 0.765 |  | 0.999 | 0.984 |
| SFG | 0.995 | 0.995 |  | 0.998 | 0.994 |  | 0.313 | 0.367 |  | 0.568 | 0.564 |  | 0.859 | 0.918 |  | 0.956 | 0.926 |
| PMd | 0.995 | 0.995 |  | 0.998 | 0.994 |  | 0.460 | 0.538 |  | 0.568 | 0.564 |  | 0.778 | 0.771 |  | 0.983 | 0.974 |
| PMv | 0.995 | 0.995 |  | 0.998 | 0.994 |  | 0.313 | 0.393 |  | 0.737 | 0.618 |  | 0.710 | 0.765 |  | 0.999 | 0.974 |
| preSMA | 0.995 | 0.995 |  | 0.998 | 0.994 |  | 0.313 | 0.393 |  | 0.568 | 0.564 |  | 0.857 | 0.936 |  | 0.956 | 0.926 |
| M1 | 0.995 | 0.995 |  | 0.998 | 0.994 |  | 0.313 | 0.393 |  | 0.568 | 0.564 |  | 0.804 | 0.771 |  | 0.999 | 0.974 |
| SMA | 0.995 | 0.995 |  | 0.998 | 0.994 |  | 0.415 | 0.483 |  | 0.568 | 0.564 |  | 0.804 | 0.805 |  | 0.956 | 0.926 |
| ITG | 0.995 | 0.995 |  | 0.998 | 0.994 |  | 0.347 | 0.447 |  | 0.568 | 0.564 |  | 0.778 | 0.771 |  | 0.956 | 0.926 |
| MTG | 0.995 | 0.995 |  | 0.998 | 0.994 |  | 0.313 | 0.393 |  | 0.589 | 0.564 |  | 0.710 | 0.765 |  | 0.956 | 0.926 |
| STG | 0.995 | 0.995 |  | 0.998 | 0.994 |  | 0.275 | 0.367 |  | 0.568 | 0.564 |  | 0.710 | 0.765 |  | 0.983 | 0.926 |
| S1 | 0.995 | 0.995 |  | 0.998 | 0.994 |  | 0.275 | 0.367 |  | 0.793 | 0.798 |  | 0.859 | 0.971 |  | 0.999 | 0.974 |
| PCL | 0.995 | 0.995 |  | 0.998 | 0.994 |  | 0.290 | 0.367 |  | 0.568 | 0.564 |  | 0.778 | 0.765 |  | 0.999 | 0.974 |
| IPL | 0.995 | 0.995 |  | 0.998 | 0.994 |  | 0.196 | 0.367 |  | 0.568 | 0.564 |  | 0.710 | 0.765 |  | 0.956 | 0.926 |
| SPL | 0.995 | 0.995 |  | 0.998 | 0.994 |  | 0.265 | 0.367 |  | 0.568 | 0.564 |  | 0.804 | 0.855 |  | 0.956 | 0.926 |
| SMG | 0.995 | 0.995 |  | 0.998 | 0.994 |  | 0.265 | 0.367 |  | 0.568 | 0.564 |  | 0.804 | 0.765 |  | 0.956 | 0.926 |
| ANG | 0.995 | 0.995 |  | 0.998 | 0.994 |  | 0.265 | 0.367 |  | 0.589 | 0.564 |  | 0.778 | 0.765 |  | 0.956 | 0.926 |
| CAL | 0.995 | 0.995 |  | 0.998 | 0.994 |  | 0.341 | 0.421 |  | 0.742 | 0.692 |  | 0.778 | 0.765 |  | 0.999 | 0.974 |
| CUN | 0.995 | 0.995 |  | 0.998 | 0.994 |  | 0.313 | 0.393 |  | 0.568 | 0.564 |  | 0.710 | 0.765 |  | 0.956 | 0.937 |
| LIG | 0.995 | 0.995 |  | 0.998 | 0.994 |  | 0.275 | 0.367 |  | 0.568 | 0.564 |  | 0.710 | 0.765 |  | 0.999 | 0.974 |
| IOG | 0.995 | 0.995 |  | 0.998 | 0.994 |  | 0.313 | 0.393 |  | 0.568 | 0.564 |  | 0.857 | 0.936 |  | 0.999 | 0.974 |
| MOG | 0.995 | 0.995 |  | 0.998 | 0.994 |  | 0.313 | 0.393 |  | 0.568 | 0.564 |  | 0.778 | 0.765 |  | 0.956 | 0.926 |
| SOG | 0.995 | 0.995 |  | 0.998 | 0.994 |  | 0.284 | 0.367 |  | 0.568 | 0.564 |  | 0.710 | 0.765 |  | 0.956 | 0.926 |

**Supplementary Table 11.** Nonparametric partial correlation results between free water in white matter and clinical measures of ASD

|  | RBS-R total raw score | | |  | ADOS-2 total raw score | | |
| --- | --- | --- | --- | --- | --- | --- | --- |
| ROI | R | *p*_raw_ | *p*_FDR_ |  | R | *p*_raw_ | *p*_FDR_ |
| GR | -0.029 | 0.890 | 0.975 |  | -0.202 | **0.041** | 0.072 |
| mOG | -0.077 | 0.651 | 0.911 |  | -0.192 | 0.057 | 0.080 |
| OC | -0.007 | 0.975 | 0.975 |  | -0.202 | **0.034** | 0.072 |
| PMd | -0.162 | 0.339 | 0.593 |  | -0.247 | **0.038** | 0.072 |
| PMv | -0.174 | 0.226 | 0.593 |  | -0.173 | **0.032** | 0.072 |
| preSMA | -0.157 | 0.336 | 0.593 |  | -0.194 | 0.123 | 0.143 |
| SMA | -0.190 | 0.296 | 0.593 |  | -0.155 | 0.273 | 0.273 |

**Supplementary Table 12.** Fractional anisotropy (FA) of 32 transcallosal tracts in autistic adults (ASD) and neurotypical controls (NT) and ANCOVA **results between the two groups**

|  | ASD | |  | NT | |  | η*_p_*^2^ |  | *p*_raw_ |  | *p*_FDR_ |
| --- | --- | --- | --- | --- | --- | --- | --- | --- | --- | --- | --- |
| ROIs | Mean | SD |  | Mean | SD |  |  |  |  |  |  |
| AOG | 0.496 | 0.0301 |  | 0.496 | 0.0373 |  | 0.006 |  | 0.503 |  | 0.616 |
| GR | 0.395 | 0.0255 |  | 0.398 | 0.0349 |  | 0.017 |  | 0.232 |  | 0.511 |
| IFG_oper | 0.422 | 0.0239 |  | 0.421 | 0.0339 |  | 0.004 |  | 0.583 |  | 0.628 |
| IFG_orb | 0.474 | 0.0235 |  | 0.476 | 0.0332 |  | 0.012 |  | 0.322 |  | 0.511 |
| IFG_tri | 0.384 | 0.0247 |  | 0.385 | 0.0308 |  | 0.010 |  | 0.355 |  | 0.527 |
| LOG | 0.505 | 0.0245 |  | 0.509 | 0.0341 |  | 0.021 |  | 0.188 |  | 0.511 |
| mFG | 0.456 | 0.0258 |  | 0.458 | 0.0365 |  | 0.013 |  | 0.311 |  | 0.511 |
| mOG | 0.459 | 0.0300 |  | 0.461 | 0.0396 |  | 0.015 |  | 0.266 |  | 0.511 |
| mOFG | 0.438 | 0.0270 |  | 0.441 | 0.0345 |  | 0.019 |  | 0.208 |  | 0.511 |
| MFG | 0.385 | 0.0227 |  | 0.386 | 0.0312 |  | 0.012 |  | 0.324 |  | 0.511 |
| OC | 0.355 | 0.0223 |  | 0.358 | 0.0316 |  | 0.019 |  | 0.212 |  | 0.511 |
| SFG | 0.435 | 0.0243 |  | 0.437 | 0.0352 |  | 0.013 |  | 0.306 |  | 0.511 |
| PMd | 0.413 | 0.0197 |  | 0.415 | 0.0312 |  | 0.013 |  | 0.305 |  | 0.511 |
| PMv | 0.405 | 0.0204 |  | 0.408 | 0.0306 |  | 0.020 |  | 0.200 |  | 0.511 |
| preSMA | 0.452 | 0.0241 |  | 0.458 | 0.0383 |  | 0.030 |  | 0.118 |  | 0.511 |
| M1 | 0.438 | 0.0199 |  | 0.438 | 0.0299 |  | 0.005 |  | 0.512 |  | 0.616 |
| SMA | 0.438 | 0.0233 |  | 0.439 | 0.0327 |  | 0.010 |  | 0.375 |  | 0.538 |
| ITG | 0.465 | 0.0208 |  | 0.466 | 0.0251 |  | 0.003 |  | 0.596 |  | 0.638 |
| MTG | 0.489 | 0.0222 |  | 0.488 | 0.0267 |  | <0.001 |  | 0.885 |  | 0.919 |
| STG | 0.441 | 0.0213 |  | 0.439 | 0.0291 |  | <0.001 |  | 0.999 |  | 0.999 |
| S1 | 0.444 | 0.0217 |  | 0.445 | 0.0307 |  | 0.005 |  | 0.523 |  | 0.616 |
| PCL | 0.417 | 0.0256 |  | 0.419 | 0.0314 |  | 0.013 |  | 0.296 |  | 0.511 |
| IPL | 0.433 | 0.0212 |  | 0.437 | 0.0272 |  | 0.021 |  | 0.191 |  | 0.511 |
| SPL | 0.476 | 0.0236 |  | 0.478 | 0.0267 |  | 0.004 |  | 0.546 |  | 0.628 |
| SMG | 0.432 | 0.0185 |  | 0.434 | 0.0262 |  | 0.009 |  | 0.387 |  | 0.538 |
| ANG | 0.446 | 0.0218 |  | 0.450 | 0.0309 |  | 0.017 |  | 0.243 |  | 0.511 |
| CAL | 0.470 | 0.0258 |  | 0.477 | 0.0354 |  | 0.023 |  | 0.169 |  | 0.511 |
| CUN | 0.517 | 0.0265 |  | 0.520 | 0.0331 |  | 0.007 |  | 0.441 |  | 0.604 |
| LIG | 0.446 | 0.0247 |  | 0.453 | 0.0337 |  | 0.028 |  | 0.125 |  | 0.511 |
| IOG | 0.519 | 0.0279 |  | 0.528 | 0.0350 |  | 0.039 |  | 0.074 |  | 0.511 |
| MOG | 0.491 | 0.0260 |  | 0.497 | 0.0321 |  | 0.020 |  | 0.202 |  | 0.511 |
| SOG | 0.519 | 0.0280 |  | 0.525 | 0.0331 |  | 0.017 |  | 0.240 |  | 0.511 |

**Supplementary Table 13.** Mean diffusivity (MD) of 32 transcallosal tracts in autistic adults (ASD) and neurotypical controls (NT) and ANCOVA **results between the two groups**

|  | ASD | |  | NT | |  | η*_p_*^2^ |  | *p*_raw_ |  | *p*_FDR_ |
| --- | --- | --- | --- | --- | --- | --- | --- | --- | --- | --- | --- |
| ROIs | Mean | SD |  | Mean | SD |  |  |  |  |  |  |
| AOG | 8.33E-04 | 3.97E-05 |  | 8.25E-04 | 4.30E-05 |  | 0.023 |  | 0.173 |  | 0.300 |
| GR | 8.02E-04 | 3.77E-05 |  | 7.86E-04 | 3.04E-05 |  | 0.065 |  | **0.019** |  | 0.107 |
| IFG_oper | 7.94E-04 | 3.71E-05 |  | 7.83E-04 | 4.32E-05 |  | 0.049 |  | **0.043** |  | 0.181 |
| IFG_orb | 8.20E-04 | 3.61E-05 |  | 8.12E-04 | 4.39E-05 |  | 0.026 |  | 0.142 |  | 0.281 |
| IFG_tri | 7.78E-04 | 3.23E-05 |  | 7.70E-04 | 4.10E-05 |  | 0.030 |  | 0.117 |  | 0.275 |
| LOG | 8.30E-04 | 3.83E-05 |  | 8.22E-04 | 4.16E-05 |  | 0.030 |  | 0.117 |  | 0.275 |
| mFG | 7.86E-04 | 3.21E-05 |  | 7.78E-04 | 5.51E-05 |  | 0.016 |  | 0.255 |  | 0.367 |
| mOG | 8.43E-04 | 4.63E-05 |  | 8.27E-04 | 3.89E-05 |  | 0.060 |  | **0.024** |  | 0.123 |
| mOFG | 7.95E-04 | 3.28E-05 |  | 7.84E-04 | 4.15E-05 |  | 0.032 |  | 0.103 |  | 0.275 |
| MFG | 8.03E-04 | 3.41E-05 |  | 7.94E-04 | 4.59E-05 |  | 0.030 |  | 0.114 |  | 0.275 |
| OC | 7.91E-04 | 3.49E-05 |  | 7.75E-04 | 2.90E-05 |  | 0.072 |  | **0.014** |  | 0.096 |
| SFG | 7.79E-04 | 3.12E-05 |  | 7.72E-04 | 5.27E-05 |  | 0.015 |  | 0.270 |  | 0.369 |
| PMd | 7.87E-04 | 2.75E-05 |  | 7.73E-04 | 3.44E-05 |  | 0.093 |  | **0.005** |  | 0.096 |
| PMv | 8.07E-04 | 3.56E-05 |  | 7.92E-04 | 4.04E-05 |  | 0.071 |  | **0.014** |  | 0.096 |
| preSMA | 7.97E-04 | 3.03E-05 |  | 7.82E-04 | 3.88E-05 |  | 0.085 |  | **0.007** |  | 0.096 |
| M1 | 7.92E-04 | 2.63E-05 |  | 7.83E-04 | 3.54E-05 |  | 0.040 |  | 0.070 |  | 0.250 |
| SMA | 8.03E-04 | 2.96E-05 |  | 7.89E-04 | 3.37E-05 |  | 0.090 |  | **0.006** |  | 0.096 |
| ITG | 8.41E-04 | 3.44E-05 |  | 8.31E-04 | 3.46E-05 |  | 0.029 |  | 0.121 |  | 0.275 |
| MTG | 8.33E-04 | 3.68E-05 |  | 8.25E-04 | 3.68E-05 |  | 0.022 |  | 0.175 |  | 0.300 |
| STG | 8.06E-04 | 3.57E-05 |  | 7.99E-04 | 3.62E-05 |  | 0.018 |  | 0.223 |  | 0.325 |
| S1 | 8.27E-04 | 3.04E-05 |  | 8.20E-04 | 3.98E-05 |  | 0.027 |  | 0.134 |  | 0.275 |
| PCL | 7.96E-04 | 3.14E-05 |  | 7.90E-04 | 3.67E-05 |  | 0.023 |  | 0.164 |  | 0.300 |
| IPL | 7.99E-04 | 3.17E-05 |  | 7.92E-04 | 4.08E-05 |  | 0.020 |  | 0.196 |  | 0.314 |
| SPL | 8.10E-04 | 3.00E-05 |  | 8.08E-04 | 5.00E-05 |  | 0.004 |  | 0.560 |  | 0.672 |
| SMG | 8.10E-04 | 3.87E-05 |  | 7.99E-04 | 3.96E-05 |  | 0.035 |  | 0.090 |  | 0.275 |
| ANG | 8.02E-04 | 3.72E-05 |  | 7.99E-04 | 5.04E-05 |  | 0.005 |  | 0.504 |  | 0.621 |
| CAL | 8.76E-04 | 4.90E-05 |  | 8.72E-04 | 6.14E-05 |  | 0.003 |  | 0.617 |  | 0.679 |
| CUN | 8.12E-04 | 3.42E-05 |  | 8.13E-04 | 5.06E-05 |  | <0.001 |  | 0.974 |  | 0.971 |
| LIG | 9.19E-04 | 6.88E-05 |  | 9.04E-04 | 6.81E-05 |  | 0.018 |  | 0.227 |  | 0.325 |
| IOG | 8.81E-04 | 5.19E-05 |  | 8.72E-04 | 5.93E-05 |  | 0.010 |  | 0.356 |  | 0.440 |
| MOG | 8.17E-04 | 3.67E-05 |  | 8.15E-04 | 4.56E-05 |  | 0.003 |  | 0.633 |  | 0.679 |
| SOG | 8.32E-04 | 3.73E-05 |  | 8.31E-04 | 5.31E-05 |  | <0.001 |  | 0.795 |  | 0.830 |

**Supplementary Table 14.** Fractional anisotropy (FA) of 32 gray matter ROIs in autistic adults (ASD) and neurotypical controls (NT) and ANCOVA **results between the two groups**

|  | ASD | |  | NT | |  | η*_p_*^2^ |  | *p*_raw_ |  | *p*_FDR_ |
| --- | --- | --- | --- | --- | --- | --- | --- | --- | --- | --- | --- |
| ROIs | Mean | SD |  | Mean | SD |  |  |  |  |  |  |
| AOG | 0.274 | 0.0185 |  | 0.280 | 0.0310 |  | 0.038 |  | 0.076 |  | 0.936 |
| GR | 0.079 | 0.0104 |  | 0.079 | 0.0166 |  | 0.003 |  | 0.599 |  | 0.936 |
| IFG_oper | 0.144 | 0.0123 |  | 0.146 | 0.0236 |  | 0.007 |  | 0.446 |  | 0.936 |
| IFG_orb | 0.126 | 0.0193 |  | 0.128 | 0.0262 |  | 0.007 |  | 0.463 |  | 0.936 |
| IFG_tri | 0.149 | 0.0130 |  | 0.151 | 0.0245 |  | 0.014 |  | 0.289 |  | 0.936 |
| LOG | 0.136 | 0.0178 |  | 0.136 | 0.0275 |  | 0.002 |  | 0.679 |  | 0.936 |
| mFG | 0.171 | 0.0097 |  | 0.174 | 0.0190 |  | 0.019 |  | 0.213 |  | 0.936 |
| mOG | 0.118 | 0.0143 |  | 0.119 | 0.0208 |  | 0.005 |  | 0.537 |  | 0.936 |
| mOFG | 0.143 | 0.0117 |  | 0.146 | 0.0249 |  | 0.010 |  | 0.372 |  | 0.936 |
| MFG | 0.194 | 0.0129 |  | 0.198 | 0.0242 |  | 0.020 |  | 0.196 |  | 0.936 |
| OC | 0.113 | 0.0387 |  | 0.111 | 0.0298 |  | <0.001 |  | 0.949 |  | 0.936 |
| SFG | 0.166 | 0.0094 |  | 0.168 | 0.0192 |  | 0.017 |  | 0.243 |  | 0.936 |
| PMd | 0.142 | 0.0125 |  | 0.142 | 0.0155 |  | 0.002 |  | 0.675 |  | 0.936 |
| PMv | 0.206 | 0.0118 |  | 0.207 | 0.0179 |  | 0.010 |  | 0.354 |  | 0.936 |
| preSMA | 0.155 | 0.0147 |  | 0.154 | 0.0174 |  | 0.002 |  | 0.679 |  | 0.936 |
| M1 | 0.169 | 0.0114 |  | 0.169 | 0.0152 |  | <0.001 |  | 0.785 |  | 0.936 |
| SMA | 0.180 | 0.0154 |  | 0.178 | 0.0156 |  | <0.001 |  | 0.778 |  | 0.936 |
| ITG | 0.117 | 0.0235 |  | 0.116 | 0.0303 |  | <0.001 |  | 0.927 |  | 0.936 |
| MTG | 0.143 | 0.0210 |  | 0.143 | 0.0257 |  | <0.001 |  | 0.813 |  | 0.936 |
| STG | 0.148 | 0.0120 |  | 0.148 | 0.0219 |  | 0.002 |  | 0.660 |  | 0.936 |
| S1 | 0.152 | 0.0106 |  | 0.151 | 0.0189 |  | <0.001 |  | 0.787 |  | 0.936 |
| PCL | 0.148 | 0.0093 |  | 0.150 | 0.0169 |  | 0.011 |  | 0.335 |  | 0.936 |
| IPL | 0.221 | 0.0150 |  | 0.227 | 0.0224 |  | 0.052 |  | **0.037** |  | 0.936 |
| SPL | 0.177 | 0.0127 |  | 0.181 | 0.0358 |  | 0.009 |  | 0.382 |  | 0.936 |
| SMG | 0.168 | 0.0101 |  | 0.172 | 0.0201 |  | 0.030 |  | 0.116 |  | 0.936 |
| ANG | 0.198 | 0.0146 |  | 0.202 | 0.0313 |  | 0.012 |  | 0.327 |  | 0.936 |
| CAL | 0.133 | 0.0129 |  | 0.137 | 0.0268 |  | 0.019 |  | 0.216 |  | 0.936 |
| CUN | 0.138 | 0.0178 |  | 0.140 | 0.0488 |  | <0.001 |  | 0.811 |  | 0.936 |
| LIG | 0.108 | 0.0136 |  | 0.110 | 0.0295 |  | 0.005 |  | 0.534 |  | 0.936 |
| IOG | 0.107 | 0.0235 |  | 0.105 | 0.0412 |  | <0.001 |  | 0.904 |  | 0.936 |
| MOG | 0.140 | 0.0253 |  | 0.145 | 0.0445 |  | 0.006 |  | 0.472 |  | 0.936 |
| SOG | 0.166 | 0.0206 |  | 0.169 | 0.0591 |  | 0.003 |  | 0.644 |  | 0.936 |

**Supplementary Table 15.** Mean diffusivity (MD) of 32 gray matter ROIs in autistic adults (ASD) and neurotypical controls (NT) and ANCOVA **results between the two groups**

|  | ASD | |  | NT | |  | η*_p_*^2^ |  | *p*_raw_ |  | *p*_FDR_ |
| --- | --- | --- | --- | --- | --- | --- | --- | --- | --- | --- | --- |
| ROIs | Mean | SD |  | Mean | SD |  |  |  |  |  |  |
| AOG | 7.74E-04 | 4.16E-05 |  | 7.67E-04 | 6.62E-05 |  | 0.007 |  | 0.463 |  | 0.537 |
| GR | 1.03E-03 | 1.51E-04 |  | 9.90E-04 | 1.63E-04 |  | 0.031 |  | 0.109 |  | 0.162 |
| IFG_oper | 1.02E-03 | 9.86E-05 |  | 9.91E-04 | 1.07E-04 |  | 0.050 |  | **0.041** |  | 0.116 |
| IFG_orb | 1.02E-03 | 1.24E-04 |  | 9.95E-04 | 1.13E-04 |  | 0.023 |  | 0.172 |  | 0.226 |
| IFG_tri | 9.84E-04 | 7.92E-05 |  | 9.50E-04 | 8.35E-05 |  | 0.071 |  | **0.014** |  | 0.097 |
| LOG | 8.64E-04 | 5.84E-05 |  | 8.46E-04 | 6.14E-05 |  | 0.028 |  | 0.129 |  | 0.173 |
| mFG | 1.01E-03 | 1.02E-04 |  | 9.84E-04 | 1.11E-04 |  | 0.042 |  | 0.060 |  | 0.138 |
| mOG | 9.68E-04 | 9.38E-05 |  | 9.33E-04 | 9.61E-05 |  | 0.055 |  | **0.032** |  | 0.116 |
| mOFG | 9.87E-04 | 8.64E-05 |  | 9.53E-04 | 7.36E-05 |  | 0.060 |  | **0.025** |  | 0.097 |
| MFG | 8.75E-04 | 5.77E-05 |  | 8.67E-04 | 1.14E-04 |  | 0.007 |  | 0.435 |  | 0.532 |
| OC | 1.32E-03 | 2.15E-04 |  | 1.26E-03 | 1.79E-04 |  | 0.037 |  | 0.080 |  | 0.140 |
| SFG | 9.87E-04 | 1.00E-04 |  | 9.58E-04 | 1.02E-04 |  | 0.046 |  | 0.050 |  | 0.138 |
| PMd | 1.14E-03 | 1.49E-04 |  | 1.11E-03 | 1.06E-04 |  | 0.039 |  | 0.072 |  | 0.140 |
| PMv | 9.32E-04 | 6.49E-05 |  | 9.23E-04 | 6.26E-05 |  | 0.022 |  | 0.182 |  | 0.226 |
| preSMA | 1.19E-03 | 1.64E-04 |  | 1.15E-03 | 1.03E-04 |  | 0.036 |  | 0.086 |  | 0.140 |
| M1 | 1.07E-03 | 1.19E-04 |  | 1.06E-03 | 8.21E-05 |  | 0.016 |  | 0.245 |  | 0.283 |
| SMA | 1.11E-03 | 1.34E-04 |  | 1.09E-03 | 8.66E-05 |  | 0.018 |  | 0.230 |  | 0.283 |
| ITG | 8.09E-04 | 7.25E-05 |  | 7.80E-04 | 5.68E-05 |  | 0.067 |  | **0.017** |  | 0.097 |
| MTG | 8.35E-04 | 3.42E-05 |  | 8.25E-04 | 3.55E-05 |  | 0.038 |  | 0.075 |  | 0.140 |
| STG | 1.01E-03 | 7.01E-05 |  | 9.88E-04 | 8.05E-05 |  | 0.053 |  | **0.035** |  | 0.116 |
| S1 | 1.07E-03 | 1.19E-04 |  | 1.08E-03 | 9.13E-05 |  | 0.001 |  | 0.728 |  | 0.735 |
| PCL | 1.02E-03 | 1.06E-04 |  | 9.99E-04 | 8.77E-05 |  | 0.035 |  | 0.089 |  | 0.140 |
| IPL | 8.26E-04 | 3.88E-05 |  | 8.16E-04 | 5.06E-05 |  | 0.035 |  | 0.087 |  | 0.140 |
| SPL | 9.87E-04 | 8.28E-05 |  | 9.74E-04 | 1.11E-04 |  | 0.013 |  | 0.308 |  | 0.342 |
| SMG | 9.03E-04 | 5.73E-05 |  | 8.89E-04 | 7.27E-05 |  | 0.043 |  | 0.060 |  | 0.138 |
| ANG | 8.41E-04 | 4.18E-05 |  | 8.32E-04 | 4.54E-05 |  | 0.032 |  | 0.104 |  | 0.153 |
| CAL | 9.53E-04 | 6.29E-05 |  | 9.18E-04 | 6.67E-05 |  | 0.109 |  | **0.002** |  | 0.064 |
| CUN | 9.02E-04 | 5.36E-05 |  | 8.83E-04 | 4.76E-05 |  | 0.076 |  | **0.011** |  | 0.097 |
| LIG | 1.11E-03 | 1.11E-04 |  | 1.06E-03 | 9.71E-05 |  | 0.067 |  | **0.018** |  | 0.097 |
| IOG | 8.54E-04 | 8.94E-05 |  | 8.16E-04 | 7.69E-05 |  | 0.065 |  | **0.019** |  | 0.097 |
| MOG | 8.40E-04 | 5.58E-05 |  | 8.22E-04 | 6.58E-05 |  | 0.035 |  | 0.088 |  | 0.140 |
| SOG | 8.71E-04 | 6.00E-05 |  | 8.45E-04 | 5.80E-05 |  | 0.062 |  | **0.022** |  | 0.097 |

**Supplementary Table 16.** Nonparametric partial correlation results between free water uncorrected diffusion measures (FA and MD) across 32 transcallosal tracts and age in autistic adults (ASD) and neurotypical controls (NT)

|  |  | FA | | | | |  | MD | | | | |
| --- | --- | --- | --- | --- | --- | --- | --- | --- | --- | --- | --- | --- |
|  |  | ASD | |  | NT | |  | ASD | |  | NT | |
| ROI |  | R | *p*_FDR_ |  | R | *p*_FDR_ |  | R | *p*_FDR_ |  | R | *p*_FDR_ |
| AOG |  | -0.282 | 0.135 |  | -0.697 | **<0.001** |  | 0.241 | 0.354 |  | 0.478 | **<0.001** |
| GR |  | -0.385 | **0.033** |  | -0.702 | **<0.001** |  | 0.127 | 0.633 |  | 0.408 | **0.003** |
| IFG_oper |  | -0.504 | **0.022** |  | -0.667 | **<0.001** |  | 0.325 | 0.354 |  | 0.571 | **<0.001** |
| IFG_orb |  | -0.172 | 0.326 |  | -0.700 | **<0.001** |  | 0.174 | 0.483 |  | 0.604 | **<0.001** |
| IFG_tri |  | -0.495 | **0.022** |  | -0.659 | **<0.001** |  | 0.230 | 0.380 |  | 0.530 | **<0.001** |
| LOG |  | -0.150 | 0.365 |  | -0.710 | **<0.001** |  | 0.223 | 0.354 |  | 0.580 | **<0.001** |
| mFG |  | -0.274 | 0.132 |  | -0.681 | **<0.001** |  | 0.186 | 0.483 |  | 0.390 | **<0.001** |
| mOG |  | -0.336 | 0.058 |  | -0.732 | **<0.001** |  | 0.245 | 0.354 |  | 0.549 | **<0.001** |
| mOFG |  | -0.307 | 0.095 |  | -0.690 | **<0.001** |  | 0.139 | 0.600 |  | 0.342 | **0.007** |
| MFG |  | -0.328 | 0.058 |  | -0.671 | **<0.001** |  | 0.250 | 0.354 |  | 0.537 | **<0.001** |
| OC |  | -0.385 | **0.033** |  | -0.687 | **<0.001** |  | 0.112 | 0.669 |  | 0.351 | **0.011** |
| SFG |  | -0.268 | 0.135 |  | -0.675 | **<0.001** |  | 0.173 | 0.506 |  | 0.395 | **<0.001** |
| PMd |  | -0.300 | 0.058 |  | -0.596 | **<0.001** |  | 0.317 | 0.354 |  | 0.563 | **<0.001** |
| PMv |  | -0.328 | 0.059 |  | -0.546 | **<0.001** |  | 0.344 | 0.354 |  | 0.549 | **<0.001** |
| preSMA |  | -0.346 | **0.033** |  | -0.663 | **<0.001** |  | 0.291 | 0.354 |  | 0.580 | **<0.001** |
| M1 |  | -0.272 | 0.135 |  | -0.583 | **<0.001** |  | 0.221 | 0.354 |  | 0.488 | **<0.001** |
| SMA |  | -0.319 | 0.058 |  | -0.607 | **<0.001** |  | 0.255 | 0.354 |  | 0.577 | **<0.001** |
| ITG |  | -0.239 | 0.167 |  | -0.518 | **0.002** |  | 0.144 | 0.592 |  | 0.373 | **0.007** |
| MTG |  | -0.186 | 0.326 |  | -0.547 | **<0.001** |  | 0.175 | 0.489 |  | 0.421 | **0.003** |
| STG |  | -0.151 | 0.390 |  | -0.469 | **0.002** |  | 0.114 | 0.633 |  | 0.447 | **<0.001** |
| S1 |  | -0.130 | 0.523 |  | -0.617 | **<0.001** |  | 0.290 | 0.354 |  | 0.559 | **<0.001** |
| PCL |  | -0.375 | 0.050 |  | -0.599 | **<0.001** |  | 0.204 | 0.354 |  | 0.508 | **<0.001** |
| IPL |  | -0.185 | 0.296 |  | -0.579 | **<0.001** |  | 0.185 | 0.456 |  | 0.474 | **<0.001** |
| SPL |  | -0.077 | 0.641 |  | -0.483 | **0.002** |  | 0.141 | 0.587 |  | 0.412 | **<0.001** |
| SMG |  | -0.141 | 0.390 |  | -0.596 | **<0.001** |  | 0.220 | 0.354 |  | 0.485 | **<0.001** |
| ANG |  | -0.261 | 0.095 |  | -0.503 | **0.002** |  | 0.176 | 0.483 |  | 0.423 | **0.002** |
| CAL |  | -0.160 | 0.365 |  | -0.389 | **0.009** |  | 0.059 | 0.773 |  | 0.286 | **0.032** |
| CUN |  | -0.083 | 0.602 |  | -0.419 | **0.006** |  | -0.106 | 0.669 |  | 0.314 | **0.010** |
| LIG |  | -0.160 | 0.365 |  | -0.425 | **0.006** |  | 0.072 | 0.701 |  | 0.270 | 0.066 |
| IOG |  | -0.164 | 0.365 |  | -0.422 | **0.005** |  | 0.051 | 0.792 |  | 0.294 | **0.034** |
| MOG |  | -0.117 | 0.523 |  | -0.487 | **0.002** |  | 0.012 | 0.947 |  | 0.355 | **0.008** |
| SOG |  | -0.073 | 0.641 |  | -0.465 | **0.001** |  | -0.037 | 0.856 |  | 0.318 | **0.010** |

**Supplementary Table 17.** Nonparametric partial correlation results between free water uncorrected diffusion measures (FA and MD) across 32 gray matter origin/endpoint ROIs and age in autistic adults (ASD) and neurotypical controls (NT)

|  |  | FA | | | | |  | MD | | | | |
| --- | --- | --- | --- | --- | --- | --- | --- | --- | --- | --- | --- | --- |
|  |  | ASD | |  | NT | |  | ASD | |  | NT | |
| ROI |  | R | *p*_FDR_ |  | R | *p*_FDR_ |  | R | *p*_FDR_ |  | R | *p*_FDR_ |
| AOG |  | -0.233 | 0.213 |  | -0.514 | **0.032** |  | -0.098 | 0.764 |  | 0.257 | **0.018** |
| GR |  | -0.376 | 0.153 |  | -0.363 | **0.041** |  | 0.177 | 0.353 |  | 0.417 | **0.008** |
| IFG_oper |  | -0.375 | 0.141 |  | -0.390 | **0.035** |  | 0.249 | 0.132 |  | 0.650 | **<0.001** |
| IFG_orb |  | -0.222 | 0.288 |  | -0.270 | 0.100 |  | 0.027 | 0.882 |  | 0.397 | **0.006** |
| IFG_tri |  | -0.518 | **0.047** |  | -0.293 | 0.090 |  | 0.213 | 0.251 |  | 0.504 | **<0.001** |
| LOG |  | -0.335 | 0.183 |  | -0.247 | 0.100 |  | 0.161 | 0.406 |  | 0.210 | 0.191 |
| mFG |  | -0.377 | **0.047** |  | -0.424 | **0.035** |  | 0.276 | 0.124 |  | 0.583 | **<0.001** |
| mOG |  | -0.365 | 0.166 |  | -0.272 | 0.100 |  | 0.233 | 0.239 |  | 0.450 | **0.010** |
| mOFG |  | -0.293 | 0.192 |  | -0.212 | 0.198 |  | 0.047 | 0.837 |  | 0.483 | **0.002** |
| MFG |  | -0.246 | 0.152 |  | -0.349 | **0.041** |  | 0.253 | 0.221 |  | 0.429 | **<0.001** |
| OC |  | -0.321 | 0.192 |  | -0.092 | 0.774 |  | 0.325 | 0.086 |  | 0.144 | 0.392 |
| SFG |  | -0.287 | 0.116 |  | -0.385 | **0.049** |  | 0.267 | 0.124 |  | 0.623 | **<0.001** |
| PMd |  | -0.528 | 0.066 |  | -0.354 | 0.052 |  | 0.459 | **0.041** |  | 0.533 | **<0.001** |
| PMv |  | -0.405 | **0.047** |  | -0.557 | **0.006** |  | 0.366 | 0.085 |  | 0.668 | **<0.001** |
| preSMA |  | -0.374 | 0.153 |  | -0.391 | **0.041** |  | 0.348 | 0.085 |  | 0.524 | **<0.001** |
| M1 |  | -0.348 | 0.166 |  | -0.316 | 0.090 |  | 0.336 | 0.122 |  | 0.589 | **<0.001** |
| SMA |  | -0.377 | 0.145 |  | -0.350 | 0.090 |  | 0.322 | 0.086 |  | 0.423 | **0.013** |
| ITG |  | -0.322 | 0.192 |  | -0.047 | 0.977 |  | 0.261 | 0.122 |  | 0.352 | **0.043** |
| MTG |  | -0.321 | 0.192 |  | -0.197 | 0.248 |  | 0.149 | 0.353 |  | 0.543 | **<0.001** |
| STG |  | -0.309 | 0.192 |  | -0.168 | 0.395 |  | 0.288 | 0.108 |  | 0.626 | **<0.001** |
| S1 |  | -0.415 | 0.126 |  | -0.299 | 0.089 |  | 0.466 | **0.041** |  | 0.672 | **<0.001** |
| PCL |  | -0.297 | 0.153 |  | -0.388 | 0.052 |  | 0.241 | 0.142 |  | 0.577 | **0.002** |
| IPL |  | -0.419 | **0.047** |  | -0.462 | **0.035** |  | 0.441 | **0.041** |  | 0.538 | **0.002** |
| SPL |  | -0.059 | 0.700 |  | -0.116 | 0.679 |  | 0.179 | 0.306 |  | 0.531 | **<0.001** |
| SMG |  | -0.406 | 0.066 |  | -0.389 | **0.041** |  | 0.480 | **0.041** |  | 0.651 | **<0.001** |
| ANG |  | -0.432 | **0.047** |  | -0.189 | 0.280 |  | 0.424 | **0.041** |  | 0.547 | **0.002** |
| CAL |  | -0.309 | 0.192 |  | -0.219 | 0.184 |  | 0.350 | 0.085 |  | 0.495 | **0.003** |
| CUN |  | -0.228 | 0.373 |  | -0.027 | 0.982 |  | 0.455 | **0.048** |  | 0.552 | **0.003** |
| LIG |  | -0.397 | 0.153 |  | -0.098 | 0.774 |  | 0.361 | 0.086 |  | 0.253 | 0.175 |
| IOG |  | -0.407 | 0.150 |  | 0.006 | 0.990 |  | 0.371 | **0.048** |  | 0.082 | 0.666 |
| MOG |  | -0.308 | 0.213 |  | -0.060 | 0.965 |  | 0.407 | 0.085 |  | 0.212 | 0.191 |
| SOG |  | -0.261 | 0.243 |  | -0.041 | 0.978 |  | 0.285 | 0.086 |  | 0.293 | 0.085 |

**Supplementary Table 18.** Quadratic regression results of free water uncorrected diffusion measures (FA and MD) across transcallosal tracts with age and age^2^ in autistic adults (ASD) and neurotypical controls (NT). Table shows FDR corrected p-values for age and age^2^

|  |  | FA | | | | |  | MD | | | | |
| --- | --- | --- | --- | --- | --- | --- | --- | --- | --- | --- | --- | --- |
|  |  | ASD | |  | NT | |  | ASD | |  | NT | |
| ROI |  | Age | Age^2^ |  | Age | Age^2^ |  | Age | Age^2^ |  | Age | Age^2^ |
| AOG |  | 0.214 | 0.300 |  | 0.913 | 0.993 |  | 0.588 | 0.705 |  | 0.998 | 0.975 |
| GR |  | 0.214 | 0.307 |  | 0.913 | 0.993 |  | 0.717 | 0.804 |  | 0.998 | 0.975 |
| IFG_oper |  | 0.187 | 0.300 |  | 0.913 | 0.993 |  | 0.624 | 0.804 |  | 0.998 | 0.975 |
| IFG_orb |  | 0.187 | 0.263 |  | 0.913 | 0.993 |  | 0.606 | 0.705 |  | 0.998 | 0.975 |
| IFG_tri |  | 0.106 | 0.213 |  | 0.913 | 0.993 |  | 0.707 | 0.804 |  | 0.998 | 0.975 |
| LOG |  | 0.187 | 0.239 |  | 0.913 | 0.993 |  | 0.588 | 0.705 |  | 0.998 | 0.975 |
| mFG |  | 0.222 | 0.307 |  | 0.913 | 0.993 |  | 0.717 | 0.804 |  | 0.998 | 0.975 |
| mOG |  | 0.193 | 0.297 |  | 0.913 | 0.993 |  | 0.588 | 0.705 |  | 0.998 | 0.975 |
| mOFG |  | 0.193 | 0.297 |  | 0.913 | 0.993 |  | 0.643 | 0.786 |  | 0.998 | 0.975 |
| MFG |  | 0.131 | 0.227 |  | 0.913 | 0.993 |  | 0.611 | 0.715 |  | 0.998 | 0.975 |
| OC |  | 0.187 | 0.297 |  | 0.913 | 0.993 |  | 0.864 | 0.902 |  | 0.998 | 0.975 |
| SFG |  | 0.252 | 0.333 |  | 0.913 | 0.993 |  | 0.728 | 0.804 |  | 0.998 | 0.975 |
| PMd |  | 0.172 | 0.227 |  | 0.913 | 0.993 |  | 0.624 | 0.786 |  | 0.998 | 0.975 |
| PMv |  | 0.106 | 0.213 |  | 0.913 | 0.993 |  | 0.588 | 0.705 |  | 0.998 | 0.975 |
| preSMA |  | 0.222 | 0.308 |  | 0.913 | 0.993 |  | 0.717 | 0.804 |  | 0.998 | 0.975 |
| M1 |  | 0.106 | 0.213 |  | 0.913 | 0.993 |  | 0.611 | 0.715 |  | 0.998 | 0.975 |
| SMA |  | 0.222 | 0.308 |  | 0.913 | 0.993 |  | 0.923 | 0.804 |  | 0.998 | 0.975 |
| ITG |  | 0.172 | 0.227 |  | 0.913 | 0.993 |  | 0.588 | 0.705 |  | 0.998 | 0.975 |
| MTG |  | 0.172 | 0.227 |  | 0.913 | 0.993 |  | 0.588 | 0.705 |  | 0.998 | 0.975 |
| STG |  | 0.222 | 0.300 |  | 0.949 | 0.993 |  | 0.611 | 0.705 |  | 0.998 | 0.975 |
| S1 |  | 0.106 | 0.213 |  | 0.913 | 0.993 |  | 0.588 | 0.705 |  | 0.998 | 0.975 |
| PCL |  | 0.052 | 0.122 |  | 0.913 | 0.993 |  | 0.611 | 0.715 |  | 0.998 | 0.975 |
| IPL |  | 0.172 | 0.227 |  | 0.913 | 0.993 |  | 0.588 | 0.705 |  | 0.998 | 0.975 |
| SPL |  | 0.172 | 0.227 |  | 0.913 | 0.993 |  | 0.588 | 0.705 |  | 0.998 | 0.975 |
| SMG |  | 0.236 | 0.307 |  | 0.949 | 0.993 |  | 0.588 | 0.705 |  | 0.998 | 0.975 |
| ANG |  | 0.172 | 0.227 |  | 0.913 | 0.993 |  | 0.588 | 0.705 |  | 0.998 | 0.975 |
| CAL |  | 0.172 | 0.227 |  | 0.913 | 0.993 |  | 0.588 | 0.705 |  | 0.998 | 0.984 |
| CUN |  | 0.263 | 0.308 |  | 0.913 | 0.993 |  | 0.624 | 0.705 |  | 0.998 | 0.975 |
| LIG |  | 0.182 | 0.227 |  | 0.913 | 0.993 |  | 0.588 | 0.705 |  | 0.998 | 0.975 |
| IOG |  | 0.172 | 0.227 |  | 0.913 | 0.993 |  | 0.588 | 0.705 |  | 0.998 | 0.975 |
| MOG |  | 0.193 | 0.263 |  | 0.913 | 0.993 |  | 0.588 | 0.705 |  | 0.998 | 0.975 |
| SOG |  | 0.222 | 0.297 |  | 0.913 | 0.993 |  | 0.588 | 0.705 |  | 0.998 | 0.975 |

**Supplementary Table 19.** Quadratic regression results of free water uncorrected diffusion measures (FA and MD) across gray matter origin/endpoint ROIs with age and age^2^ in autistic adults (ASD) and neurotypical controls (NT). Table shows FDR corrected p-values for age and age^2^

|  |  | FA | | | | |  | MD | | | | |
| --- | --- | --- | --- | --- | --- | --- | --- | --- | --- | --- | --- | --- |
|  |  | ASD | |  | NT | |  | ASD | |  | NT | |
| ROI |  | Age | Age^2^ |  | Age | Age^2^ |  | Age | Age^2^ |  | Age | Age^2^ |
| AOG |  | 0.287 | 0.370 |  | 0.564 | 0.666 |  | 0.924 | 0.992 |  | 0.958 | 0.949 |
| GR |  | 0.168 | 0.272 |  | 0.564 | 0.463 |  | 0.924 | 0.992 |  | 0.598 | 0.949 |
| IFG_oper |  | 0.102 | 0.205 |  | 0.564 | 0.463 |  | 0.924 | 0.992 |  | 0.958 | 0.949 |
| IFG_orb |  | 0.251 | 0.324 |  | 0.616 | 0.481 |  | 0.924 | 0.992 |  | 0.958 | 0.949 |
| IFG_tri |  | 0.087 | 0.205 |  | 0.564 | 0.463 |  | 0.924 | 0.992 |  | 0.958 | 0.949 |
| LOG |  | 0.183 | 0.275 |  | 0.564 | 0.463 |  | 0.924 | 0.992 |  | 0.958 | 0.949 |
| mFG |  | 0.186 | 0.296 |  | 0.564 | 0.463 |  | 0.924 | 0.992 |  | 0.958 | 0.949 |
| mOG |  | 0.168 | 0.272 |  | 0.564 | 0.463 |  | 0.924 | 0.992 |  | 0.958 | 0.949 |
| mOFG |  | 0.168 | 0.272 |  | 0.564 | 0.463 |  | 0.924 | 0.992 |  | 0.958 | 0.949 |
| MFG |  | 0.102 | 0.205 |  | 0.853 | 0.666 |  | 0.924 | 0.992 |  | 0.958 | 0.949 |
| OC |  | 0.144 | 0.213 |  | 0.564 | 0.463 |  | 0.924 | 0.992 |  | 0.958 | 0.949 |
| SFG |  | 0.189 | 0.281 |  | 0.564 | 0.463 |  | 0.924 | 0.992 |  | 0.958 | 0.949 |
| PMd |  | 0.094 | 0.205 |  | 0.564 | 0.463 |  | 0.924 | 0.992 |  | 0.958 | 0.949 |
| PMv |  | 0.168 | 0.272 |  | 0.670 | 0.474 |  | 0.924 | 0.992 |  | 0.958 | 0.949 |
| preSMA |  | 0.107 | 0.205 |  | 0.564 | 0.463 |  | 0.924 | 0.992 |  | 0.958 | 0.983 |
| M1 |  | 0.107 | 0.205 |  | 0.644 | 0.484 |  | 0.924 | 0.992 |  | 0.958 | 0.949 |
| SMA |  | 0.168 | 0.272 |  | 0.564 | 0.463 |  | 0.924 | 0.992 |  | 0.958 | 0.949 |
| ITG |  | 0.168 | 0.272 |  | 0.564 | 0.481 |  | 0.924 | 0.992 |  | 0.958 | 0.949 |
| MTG |  | 0.168 | 0.272 |  | 0.564 | 0.474 |  | 0.924 | 0.992 |  | 0.958 | 0.949 |
| STG |  | 0.114 | 0.206 |  | 0.564 | 0.463 |  | 0.924 | 0.992 |  | 0.958 | 0.949 |
| S1 |  | 0.087 | 0.205 |  | 0.655 | 0.505 |  | 0.924 | 0.992 |  | 0.958 | 0.949 |
| PCL |  | 0.144 | 0.213 |  | 0.564 | 0.463 |  | 0.924 | 0.992 |  | 0.958 | 0.949 |
| IPL |  | 0.102 | 0.205 |  | 0.564 | 0.463 |  | 0.924 | 0.992 |  | 0.958 | 0.949 |
| SPL |  | 0.168 | 0.213 |  | 0.564 | 0.463 |  | 0.924 | 0.992 |  | 0.958 | 0.949 |
| SMG |  | 0.114 | 0.213 |  | 0.564 | 0.463 |  | 0.963 | 0.992 |  | 0.990 | 0.949 |
| ANG |  | 0.094 | 0.205 |  | 0.564 | 0.463 |  | 0.924 | 0.992 |  | 0.958 | 0.949 |
| CAL |  | 0.168 | 0.272 |  | 0.670 | 0.567 |  | 0.924 | 0.992 |  | 0.958 | 0.995 |
| CUN |  | 0.186 | 0.272 |  | 0.564 | 0.463 |  | 0.924 | 0.992 |  | 0.958 | 0.949 |
| LIG |  | 0.102 | 0.205 |  | 0.564 | 0.463 |  | 0.924 | 0.992 |  | 0.958 | 0.985 |
| IOG |  | 0.168 | 0.272 |  | 0.564 | 0.463 |  | 0.924 | 0.992 |  | 0.958 | 0.949 |
| MOG |  | 0.168 | 0.272 |  | 0.564 | 0.463 |  | 0.924 | 0.992 |  | 0.958 | 0.949 |
| SOG |  | 0.168 | 0.272 |  | 0.564 | 0.463 |  | 0.924 | 0.992 |  | 0.958 | 0.949 |
